# Evaluating patterns and drivers of spatial change in the recreational guided fishing sector in Alaska

**DOI:** 10.1371/journal.pone.0179584

**Published:** 2017-06-20

**Authors:** Maggie N. Chan, Anne H. Beaudreau, Philip A. Loring

**Affiliations:** 1 College of Fisheries and Ocean Sciences, University of Alaska Fairbanks, Juneau, Alaska, United States of America; 2 School of Environment and Sustainability, University of Saskatchewan, Saskatoon, Saskatchewan, Canada; Sveriges lantbruksuniversitet, SWEDEN

## Abstract

Understanding the impacts of recreational fishing on habitats and species, as well as the social and ecological importance of place to anglers, requires information on the spatial distribution of fishing activities. This study documented long-term changes in core fishing areas of a major recreational fishery in Alaska and identified biological, regulatory, social, and economic drivers of spatial fishing patterns by charter operators. Using participatory mapping and in-person interviews, we characterized the spatial footprint of 46 charter operators in the communities of Sitka and Homer since the 1990s. The spatial footprint differed between Homer and Sitka respondents, with Homer operators consistently using larger areas for Pacific halibut than Sitka operators. Homer and Sitka showed opposite trends in core fishing location area over time, with an overall decrease in Homer and an overall increase in Sitka. For both Sitka and Homer respondents, the range of areas fished was greater for Pacific halibut than for rockfish/lingcod or Pacific salmon. Spatial patterns were qualitatively different between businesses specializing in single species trips and those that operated multispecies trips and between businesses with one vessel and those with multiple vessels. In Homer, the most frequently cited reasons for changes in the location and/or extent of fishing were changes in trip type and the price of fuel, while in Sitka, the most frequently cited reasons for spatial shifts were changes to Pacific halibut regulations and gaining experience or exploring new locations. The diversity of charter fishing strategies in Alaska may allow individual charter operators to respond differently to perturbations and thus maintain resilience of the industry as a whole to social, environmental, and regulatory change. This research also highlights the importance of understanding fishers’ diverse portfolio of activities to effective ecosystem-based management.

## Introduction

Recreational fishing contributes to food security, tourism, and other economic activities in coastal communities around the world. In the United States, a 2015 national saltwater recreational fisheries policy recognized the social, cultural, and economic importance of recreational fishing and the need for improved governance of this growing sector [1]. The principles of governance outlined in this policy place particular focus on maintaining environmental sustainability and access to fishing [1]. Therefore, the success of the policy relies on information about factors affecting anglers’ access to and use of particular areas. While agencies often conduct angler surveys to document numbers, species, and sizes of harvested fish, there is rarely information on where harvest occurs. To understand the impacts of recreational fishing on habitats and species, as well as the importance of place to anglers, information is needed on the spatial distribution of fishing activities.

A wide range of biological, regulatory, economic, and cultural factors can influence where anglers choose to fish. Locations may be selected as a conservation measure [2], territoriality [3, 4], or to make political statements [5]. In addition, fish abundance has been shown to be positively correlated with angler catch rates [6] and this relationship has been explored as a driver of site selection in commercial fishing [7, 8]. However, fish abundance as a predictor of fishing location becomes problematic for small-scale fisheries because participants are not consistently motivated by high catch rates [9, 10]. In recreational fisheries, the drivers for selecting fishing locations result from the interaction of diverse factors, including economic variables, regulatory constraints, environmental conditions, and social interactions [11, 12]. In the U.S., a 2013 nationwide survey of over 9,000 recreational anglers reported that most important part of a fishing trip was spending time with family or friends (87% of responses), catching fish (83% of responses), and fishing in an uncongested area (79% of responses) [13]. In recreational charter fisheries, captains may modify aspects of the fishing experience to maintain the satisfaction of customers paying for guided fishing trips [14]. This includes providing sightseeing opportunities or offering different types of fishing trips (e.g., targeting different species, [15]), which may affect where charter vessels fish.

In this study, we examined the complex factors affecting the spatial distribution of fishing in a major United States recreational charter fishery. In Alaska, charter and sport fisheries are important both to local tourism sectors and as components of local food systems and security [16, 17]. A mail survey administered to residents and nonresidents who took sportfishing trips in Cook Inlet, Alaska, during 1997 found that for both residents and nonresidents, the primary purpose of their trip was to fish for Pacific halibut (*Hippoglossus stenolepis*) or Pacific salmon (*Oncorhynchus* spp.) [18], highlighting that access to highly-valued sportfish can be key drivers of tourism. Alaska alone harvested more sport-caught Pacific halibut (net weight) in 2015 than the combined sport fishery throughout the North American range [19]. An estimated 2,485 metric tons of Pacific halibut was harvested by Alaska’s sport sector in 2015, with approximately 53% of that from charter fishing [19], making it the most targeted bottomfish in the Alaskan charter industry (47% by number in 2014) [20]. However, declines in Pacific halibut biomass [21] have led to increased restrictions on charter halibut fishing in Alaska in the past decade, including reductions in bag and size limits [22].

**Fig 1:**
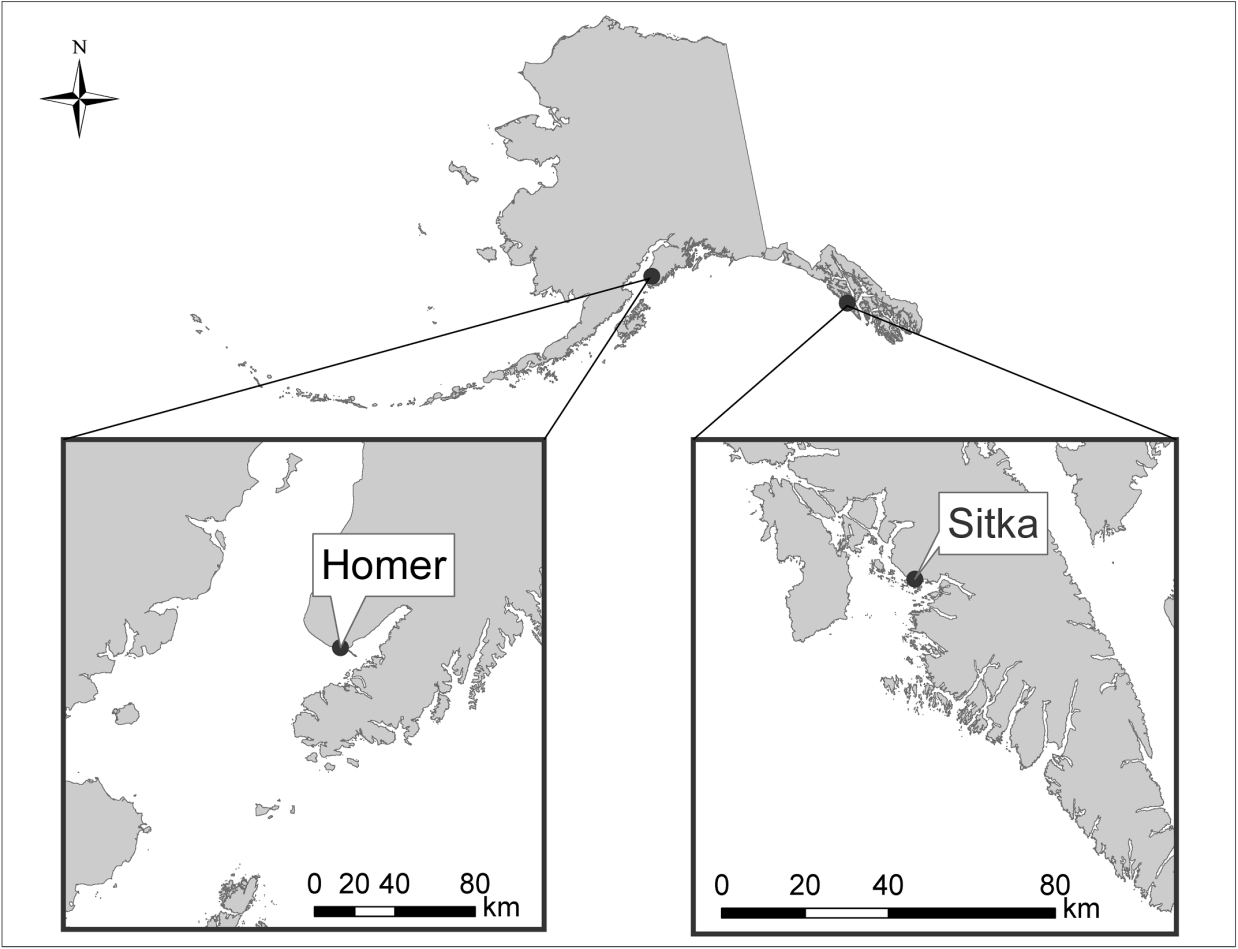
Map displaying the location of Homer and Sitka within the state of Alaska.

More than 90% of charter effort (angler days) for Pacific halibut within Alaska occurs in the Southeast and Southcentral regions (Fig 1) [20]. These two regions have different charter customer demographics and histories of halibut regulation. Participation by non-resident anglers is higher in Southeast Alaska (97% non-resident angler-days in 2014) compared to Southcentral Alaska (74% non-resident angler-days in 2014) [20]. This is a substantial increase from twenty years prior, when in 1994, 52% and 33% of angler-days fished were by non-residents in Southeast Alaska and Southcentral Alaska, respectively [23]. The relative decline of resident participation may partially be attributed to residents being more sensitive to catch rates and trip costs [24]. In addition, Pacific halibut biomass estimates differ between the two regions [25], leading to greater restrictions in Southeast Alaska over the past decade compared to Southcentral Alaska (Table 1). In Homer, the community with the highest charter halibut landings in Southcentral Alaska [20], charter businesses operate both single species (i.e., Pacific halibut only) trips and multispecies trips. In contrast, Sitka, the highest charter halibut landings in Southeast Alaska, primarily operates multispecies trips [20].

**Table 1:** Pacific halibut charter fishing regulations for Sitka and Homer, Alaska from 1993–2016.

	Sitka (Southeast Alaska)	Homer (Southcentral Alaska)
1993^a^	• 2-fish daily bag limit per customer (no size limit)	
2003	• Guideline Harvest Level (GHL) program goes into effect	
2006	• No retention of Pacific halibut by crew	
2007	• 2-fish daily bag limit per customer (32 inches max size limit on one of the fish)	
2008	• June 1–June 9: 1-fish daily bag limit per customer (no size limit) • June 10 –Dec. 31: 2-fish daily bag limit per customer (32 inches max size limit on one of the fish)	
2009	• 1-fish daily bag limit per customer (no size limit)	
2011	• Charter Halibut Limited Access Program (limited entry) goes into effect	
2011	• 1-fish daily bag limit per customer with 37 inches max size limit	
2012	• 1-fish daily bag limit per customer with a reverse slot limit. Allowable size is ≤ 45 inches or ≥ 68 inches	
2014	• 1-fish daily bag limit per customer with a reverse slot limit. Allowable size is ≤ 45 inches or ≥ 76 inches	• 2-fish daily bag limit per customer (29 inches max size limit on one of the fish) • A vessel limit of one trip per calendar day
2014	• Catch Sharing Plan (CSP) and Guided Angler Fish (GAF) go into effect	
2015	• 1-fish daily bag limit per customer with a reverse slot limit. Allowable size is ≤ 42 inches or ≥ 80 inches	• 2-fish daily bag limit per customer (29 inch max size limit on one of the fish) • A vessel limit of one trip per calendar day • 5-fish annual limit in a calendar year on charter vessel fishing trips (does not apply to GAF halibut) • Thursday closure: charter anglers may not catch and retain halibut (except GAF) on Thursday
2016	• 1-fish daily bag limit per customer with a reverse slot limit. Allowable size is ≤ 43 inches or ≥ 80 inches	• 2-fish daily bag limit per customer (28 inch max size limit on one of the fish) • A vessel limit of one trip per calendar day • 4-fish annual limit in a calendar year on charter vessel fishing trips (does not apply to GAF halibut) • Wednesday closure: charter anglers may not catch and retain halibut (except GAF) on Wednesday

Regulations displayed are for the height of the charter season (i.e., June through August). Table shows newly added regulations or adjustments to existing regulations [26, 27]

^a^No restrictions back to 1974

This study aimed to document changes to charter fishing locations over time and identified biological, regulatory, social, and economic drivers of spatial fishing patterns in Sitka and Homer. Using participatory mapping and in-person interviews, we characterized the spatial footprint of 46 charter operators since the 1990s. We hypothesized that patterns of spatial change have been different for charter captains based out of Sitka and Homer, driven by regional differences in: 1) percentage of non-resident clientele, 2) regulatory history, and 3) species availability. Additionally, multispecies trips may have a different spatial footprint than single species trips because fishing characteristics vary based on the type and number of targeted species (e.g., habitat, depth, trip duration etc.). Therefore, we also hypothesized that species diversification has affected the locations and geographic extent of fishing, specifically that core fishing locations differ between charter operators who conduct multispecies fishing trips and those who conduct single species fishing trips.

## Materials and methods

### Participant selection and sampling frame

In 2014 and 2015, in-person interviews were conducted with 46 charter fishing operators working out of Sitka (n = 27) or Homer (n = 19). In this project, we defined a charter operator as a sport fishing guide registered with the Alaska Department of Fish & Game (ADF&G). We sought respondents who are charter operators based in Sitka or Homer during the main charter season (May–Sept), have five or more years of charter fishing experience in Alaska, target Pacific halibut on at least one charter trip per year, and/or hold a Charter Halibut Permit. Interview respondents were solicited through charter association newsletters (Alaska Charter Association, Sitka Charter Boat Operators Association, Southeast Alaska Guides Organization, and Homer Charter Association) and project information mailed to 2014 Charter Halibut Permit holders with addresses in Sitka or Homer (NOAA Fisheries Charter Halibut Permits List, https://alaskafisheries.noaa.gov/permits-licenses). Additional respondents were selected using snowball sampling [28], in which each study participant recommended other knowledgeable individuals to participate. The aim of this sampling method was to identify charter operators with diverse perspectives and experience levels; therefore, we did not aim to provide representative sampling of the charter fleet in either location. Rather, snowball sampling allowed us to identify people whose practices are indicative of broader patterns of change.

This project was approved by the University of Alaska Fairbanks Institutional Review Board (IRB project #583323–2). Written consent from each participant was obtained prior to each interview.

### Participatory mapping

Mapping methods followed previous studies using local fishers to identify fishing locations [29–32]. Participants were asked to draw their primary fishing locations during charter trips on paper maps to document changes in spatial harvest patterns [31] for Pacific halibut, Pacific salmon, and rockfishes (*Sebastes* spp.). Additionally, respondents were asked to self-identify and draw locations for a fourth species that was important to charter fishing in their area. For the additional species, participants in Homer identified lingcod (*Ophiodon elongatus*), while participants in Sitka identified both lingcod and sablefish (*Anoplopoma fimbria*). We did not include fishing locations for sablefish because this species was not targeted by Homer participants and therefore, we are unable to compare between the two locations. Following identification of current fishing locations, we asked each participant if these locations had changed over time. If the current map did not cover the total years of charter fishing experience of the participant, the individual was asked to mark past fishing locations on additional maps. This process was repeated until paper maps represented the participant’s total years of charter fishing experience. Participants were asked to provide demographic information, number of years of participation in charter fishing, the type of trips they offer (e.g., half-day, full-day, multi-day) and the number of Charter Halibut Permits the operator or business possesses.

Paper maps were generated from a Geographic Information System (GIS) based index map that included local features, such as depth contours and delineation of local restricted fishing areas. All index maps were projected in the Alaska Albers coordinate system (NAD 1983–2011 Alaska Albers, WKID: 102966, Authority: ESRI). Based on pilot interviews with knowledgeable charter captains, the map scales of 1:490,000 and 1:475,000 were determined as appropriate for Sitka and Homer, respectively. An 8 km x 8 km grid was overlaid onto each paper map so that participants who did not wish to share specific fishing locations could mark grid cells. Four out of the total 46 participants (9%) chose to use the grid system instead of drawing individual fishing locations.

## Map processing

All map processing and spatial analysis was completed in ArcMap 10.2 (ArcGIS 10.2, Environmental Systems Research Institute). Paper maps were scanned at 600 dpi and imported into ArcMap. Scanned maps were georeferenced against the index map using 3 or more ground control points per map [31]. Fishing locations were outlined and converted to vector-based polygons representing fishing locations for each respondent, species group, and time period. To standardize for individual drawing variations (e.g., dots vs. polygons), we used the ‘aggregate polygons’ tool on marked fishing locations for a respondent, species group, and time period to combine fishing locations that were within 1.5 km of each other. For analysis, lingcod and rockfishes were grouped because participants reported that these species were typically targeted using the same fishing locations. Fishing locations in the 1990s for lingcod/rockfish in Sitka were excluded because of low sample sizes of fewer than 5 respondents.

## Analysis of fishing locations

The spatial distribution of fishing locations was visualized using heat maps showing fishing locations for all respondents by species group and time period. Heat maps were created by converting fishing location polygons into raster files of 1.5 km by 1.5 km grid cells. This grid cell size was used for all species groups and was approximately the mode of the distribution of polygon area size [31]. A raster sum calculator was used to identify and count overlapping fishing locations. Heat maps display the spatial distribution of fishing locations as the percentage of respondents using each 1.5 km x 1.5 km grid cell. Core fishing locations (CFL) are identified as sites with over 40% of respondents using that location, separated by species and time period. Maps are displayed by the percentage of respondents that fished in each time period and location. Pacific halibut maps additionally display polygons representing the three ADF&G sportfish statistical fishing areas with the highest charter bottomfish effort (by number of trips) for that time period. ADF&G polygons were summarized using unpublished data from the ADF&G charter logbook program [33], which records statistical fishing areas for every charter fishing trip in Alaska. We identified the three statistical fishing areas with the highest bottomfish effort for each time period relevant to our study (i.e., 1990–1999, 2000–2004, 2005–2009, and 2010–2015). Logbook data were only available for 1998–2001 and 2006–2015; therefore, ADF&G polygons for the 1990s and early 2000s were summarized using two years of logbook data.

The spatial footprint for individual respondents was evaluated using two metrics: total area fished and number of fishing locations. Total area (km^2^) was calculated as the areal sum of fishing polygons per respondent for each species and decade. The number of fishing locations was assessed by counting the number of discrete fishing polygons per respondent for each species and decade. Boxplots were used to display the statistical distributions of fishing area and number of fishing locations across respondents. Using two-sample Kolmogorov-Smirnov tests in R (Rx64, version 2.15.2, http://www.R-project.org/), we assessed differences in the distribution of total area and number of fishing locations across decades, regions, and business types. The two-sample Kolmogorov-Smirnov test is a non-parametric test assessing if the distributions of two datasets differ significantly. Based on interviews, there is wide variation in how operators choose to fish, including those who prefer to stay close to port, travel further seasonally, tailor their travel distance to specific clients, and so forth. Therefore, assessing differences in distribution rather than using central tendency measures is a more robust way of examining differences between groups, while still accounting for the diversity in human behavior. Comparisons were made both between and within regions. In Sitka, we examined whether fishing behavior was related to the size of the fishing business by comparing attributes of fishing areas between single boat owner/operators (single-boat businesses) and lodge owners and multi-vessel owner/operators (multi-boat businesses). Individuals who self-identified as contractor or employee were grouped into the appropriate category, depending on the number of boats in the business for which they primarily worked. In Homer, we examined the relationship between fishing behavior and trip type by comparing attributes of fishing areas between respondents who operated single species trips and those who operated multispecies trips. While the majority of respondents indicated some participation in both trip types, respondents typically self-identified their business with a primary specialization in single or multispecies. Therefore, each respondent in Homer was categorized as operating multispecies or single trips based on the majority of trip types over the duration of his or her charter fishing experience.

### Characterizing drivers of spatial change

During the interviews, participants were asked to explain why their charter fishing locations or area fished had changed over time (if any). For each participant, reasons for spatial change were coded using ATLAS.ti 7 (2002–2017, ATLAS.ti Scientific Software Development GmbH). Similar drivers were grouped into categories [28] and the percentage of respondents identifying each category as a driver of spatial change was calculated. Respondents were able to self-identify more than one driver of change (i.e., percentages do not add up to 100).

## Results

**Fig 2:**
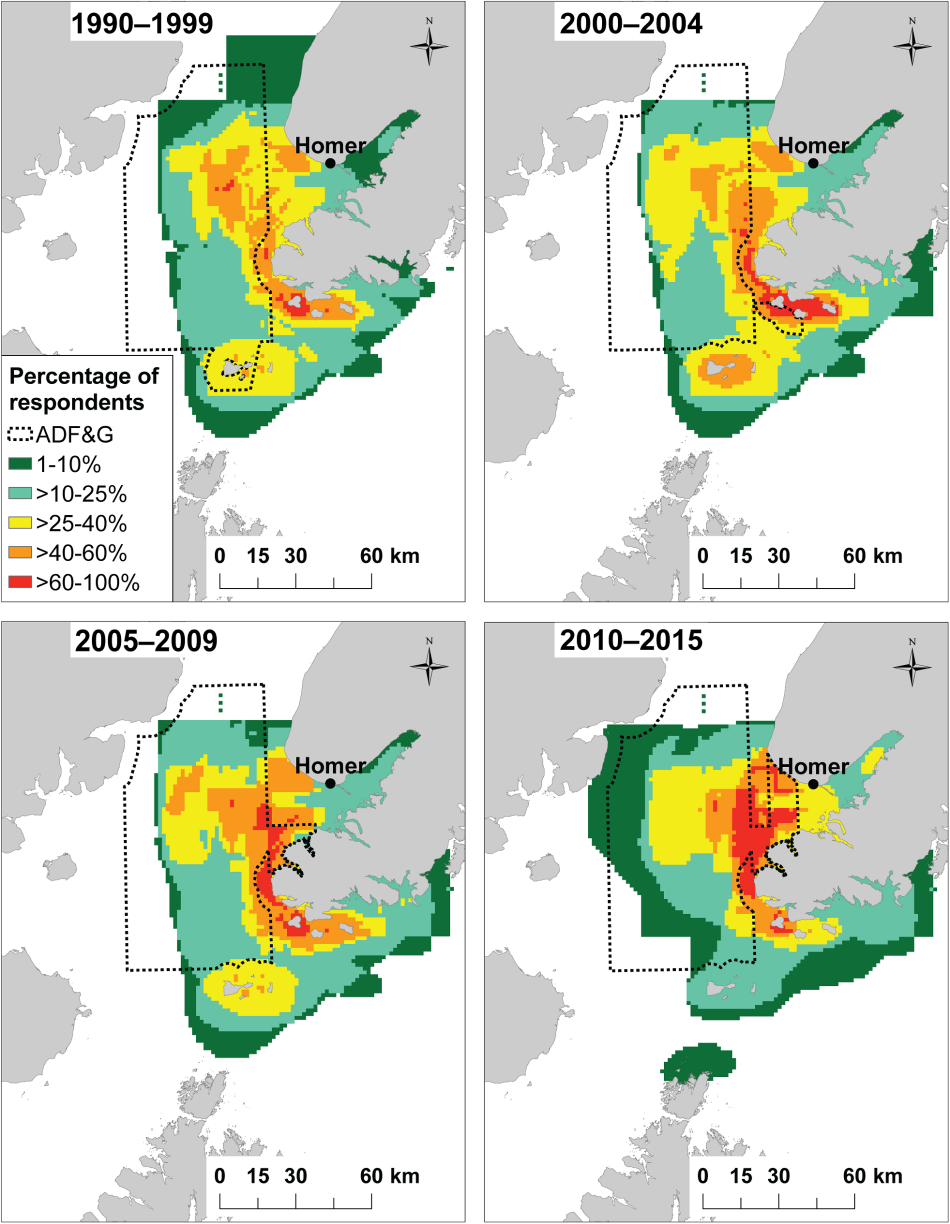
Pacific halibut fishing locations in Homer for 1990–1999, 2000–2004, 2005–2009, and 2010–2015. Locations are displayed by the percentage of respondents who fished during that time period. The dashed line represents the three ADF&G statistical fishing areas with the highest charter bottomfish effort (by number of trips), based on ADF&G charter logbook data [33].

**Fig 3:**
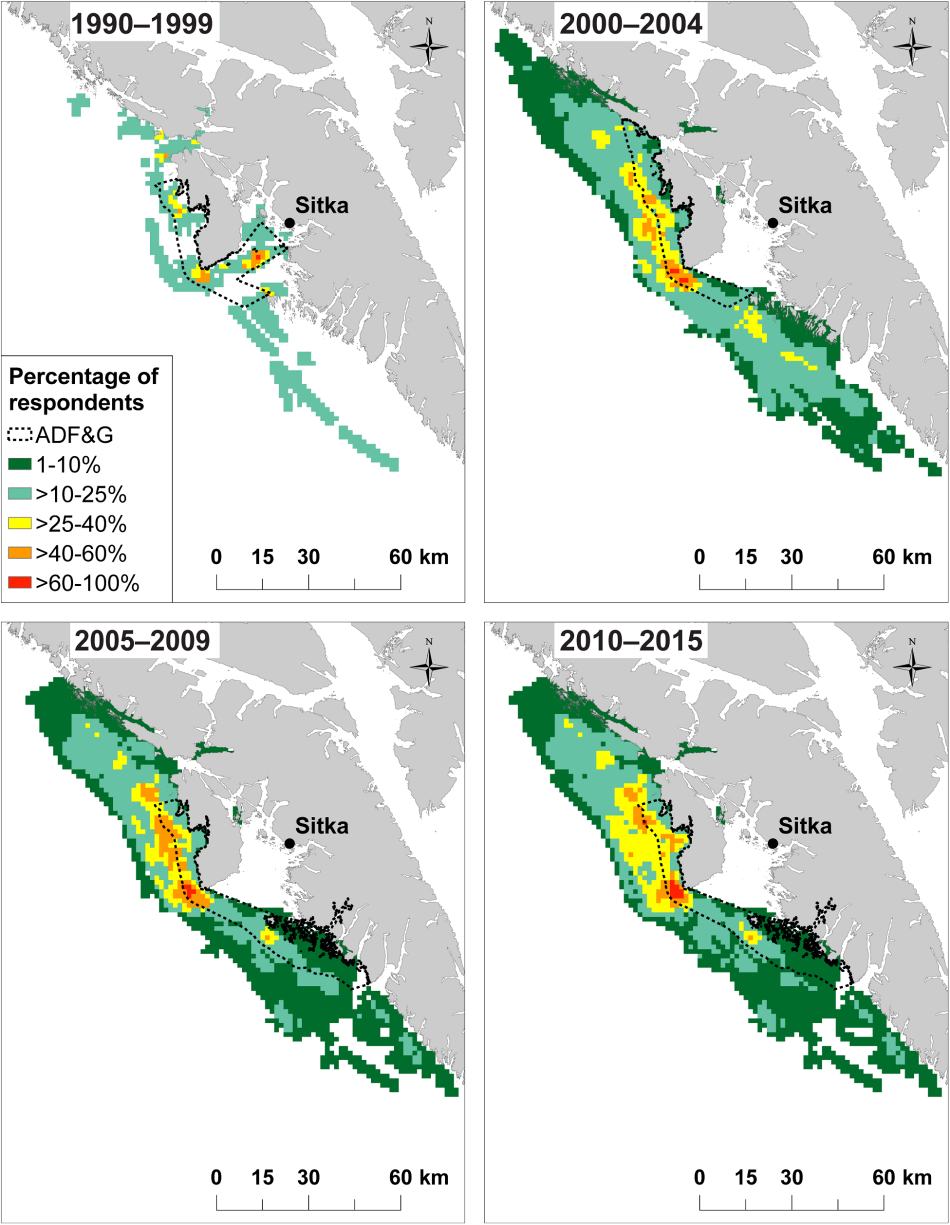
Pacific halibut fishing locations in Sitka for 1990–1999, 2000–2004, 2005–2009, and 2010–2015. Locations are displayed by the percentage of respondents who fished during that time period. The dashed line represents the three ADF&G statistical fishing areas with the highest charter bottomfish effort (by number of trips), based on ADF&G charter logbook data [33].

Comparing Homer and Sitka, there were opposite temporal trends in the spatial footprint of core fishing locations (CFL), locations where >40% of respondents target Pacific halibut. In Homer, there was an overall decrease in CFL area over time from 1,946 km^2^ in the 1990s to 1,402 km^2^ in the 2010s (Table 2). In Homer, CFL area expanded to the south in the early 2000s and subsequently retracted starting in the late 2000s (Fig 2). In contrast, for Sitka, there was an increase in CFL area over time from 43 km^2^ in the 1990s to 246 km^2^ in the 2010s (Table 2). CFL area in the 1990s was close to the port of Sitka, with just a few locations along the outer coast (Fig 3). From the early 2000s onwards, the extent of fishing expanded towards the outer coast and further north and south (Fig 3).

**Table 2:** Areas of core fishing locations for Pacific halibut by time period and town.

Town	Year	Area (km^2^)
Homer	1990–1999	1946
Homer	2000–2004	2320
Homer	2005–2009	1860
Homer	2010–2015	1402
Sitka	1990–1999	43
Sitka	2000–2004	115
Sitka	2005–2009	193
Sitka	2010–2015	246

**Fig 4:**
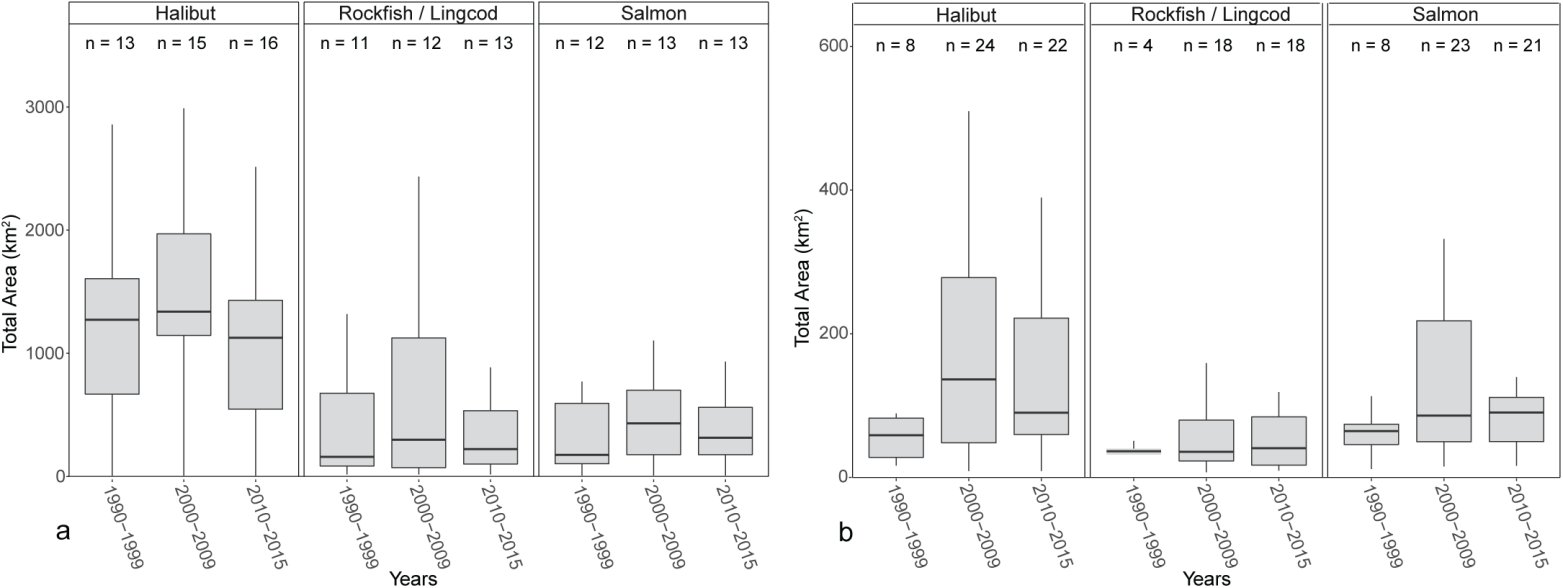
**Box and whisker plot of total fishing area (km**^**2**^**) for Pacific halibut, rockfish/lingcod, and salmon for the 1990s, 2000s, and 2010s in Homer (a) and Sitka (b).** The lower whisker extends from the first quartile to the lowest value within 1.5*IQR of the first quartile. The upper whisker extends from the third quartile to the highest value within 1.5*IQR of the third quartile. Outliers ranging above and below 1.5*IQR have been removed. Note that the scale for the y-axis is different between plots.

**Fig 5:**
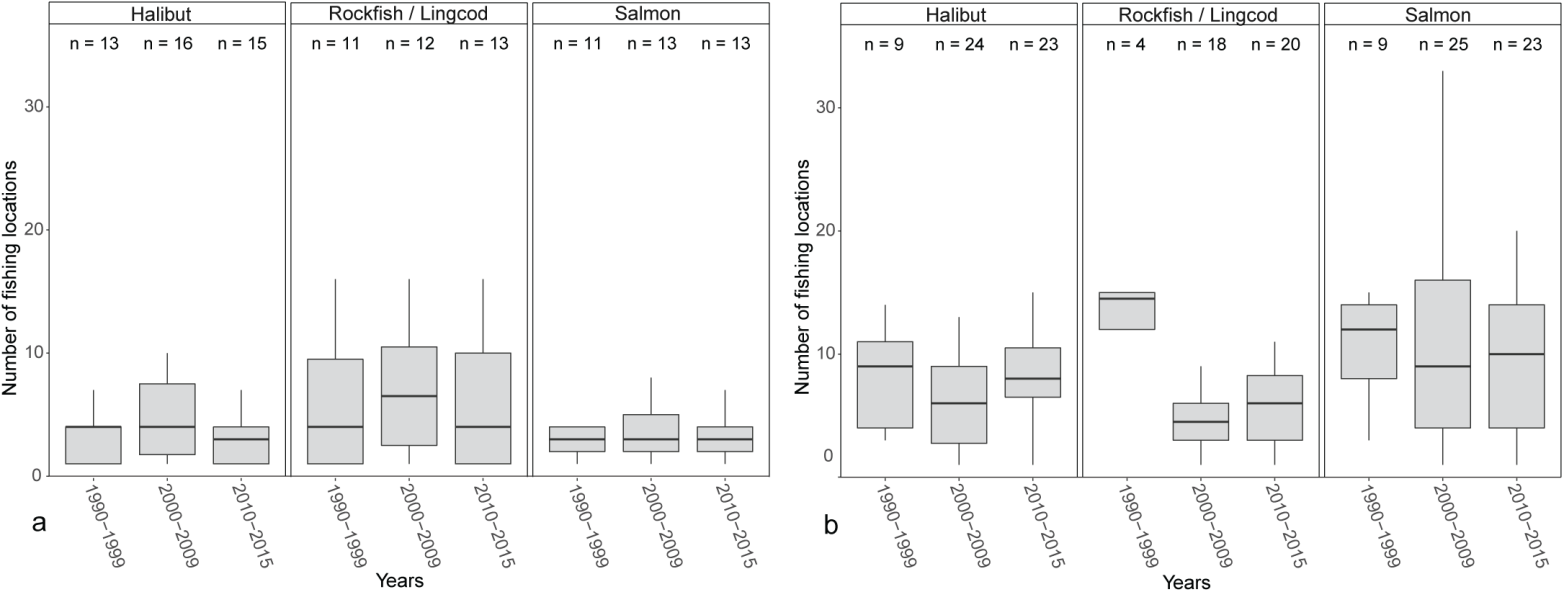
**Box and whisker plot displaying the number of fishing locations for Pacific halibut, rockfish/lingcod, and salmon for the 1990s, 2000s, and 2010s in Homer (a) and Sitka (b).** The lower whisker extends from the first quartile to the lowest value within 1.5*IQR of the first quartile. The upper whisker extends from the third quartile to the highest value within 1.5*IQR of the third quartile. Outliers ranging above and below 1.5*IQR have been removed.

In both study communities, the median and range of areas fished (km^2^) for Pacific halibut across respondents were greater than those for rockfish/lingcod or Pacific salmon (Fig 4). Median area fished for Pacific halibut was higher for charter operators in Homer compared to operators in Sitka (17 times higher in the 1990s, 10 times higher in the 2000s, and 13 times higher in the 2010s; Fig 4). The distributions of area fished for Pacific halibut differed significantly between the two communities based on a two-sample Kolmogorov-Smirnov test (Table 3). The distribution of areas fished for Pacific halibut did not differ among decades in either community, except for Sitka between the 1990s and 2000s, based on a two-sample Kolmogorov-Smirnov test (Table 4). Homer operators used fewer discrete locations for all three species groups compared to Sitka operators (Fig 5). The distribution of the number of fishing locations across respondents that were used to target Pacific halibut differed significantly between Homer and Sitka for the 2010s only (Table 3).

**Table 3:** Test statistics and p-values for two-sample Kolmogorov-Smirnov tests evaluating differences between Homer and Sitka in the distributions of total area fished and number of fishing locations across respondents.

	All time periods, all species	All time periods, halibut	1990s, halibut	2000s, halibut	2010s, halibut
Total area fished	D = 0.492, p-value = <0.01	D = 0.697, p-value = <0.01	D = 0.867, p-value = <0.01	D = 0.805, p-value = <0.01	D = 0.642, p-value = <0.01
Number of locations	D = 0.333, p-value = <0.01	D = 0.4, p-value = <0.01	D = 0.422, p-value = 0.27	D = 0.192, p-value = 0.84	D = 0.618, p-value = <0.01

**Table 4:** Test statistics and p-values for two-sample Kolmogorov-Smirnov tests evaluating differences in the distributions of total area fished and number of fishing locations across respondents for Pacific halibut.

	1990s vs. 2000s	2000s vs. 2010s	1990s vs. 2010s
**Homer**			
Total area fished	D = 0.216, p-value = 0.85	D = 0.212, p-value = 0.83	D = 0.144, p-value = 1
Number of locations	D = 0.212, p-value = 0.87	D = 0.190, p-value = 0.91	D = 0.1, p-value = 1
**Sitka**			
Total area fished	D = 0.504, p-value = 0.05	D = 0.114, p-value = 1	D = 0.489, p-value = 0.06
Number of locations	D = 0.231, p-value = 0.87	D = 0.303, p-value = 0.19	D = 0.213, p-value = 0.92

**Fig 6:**
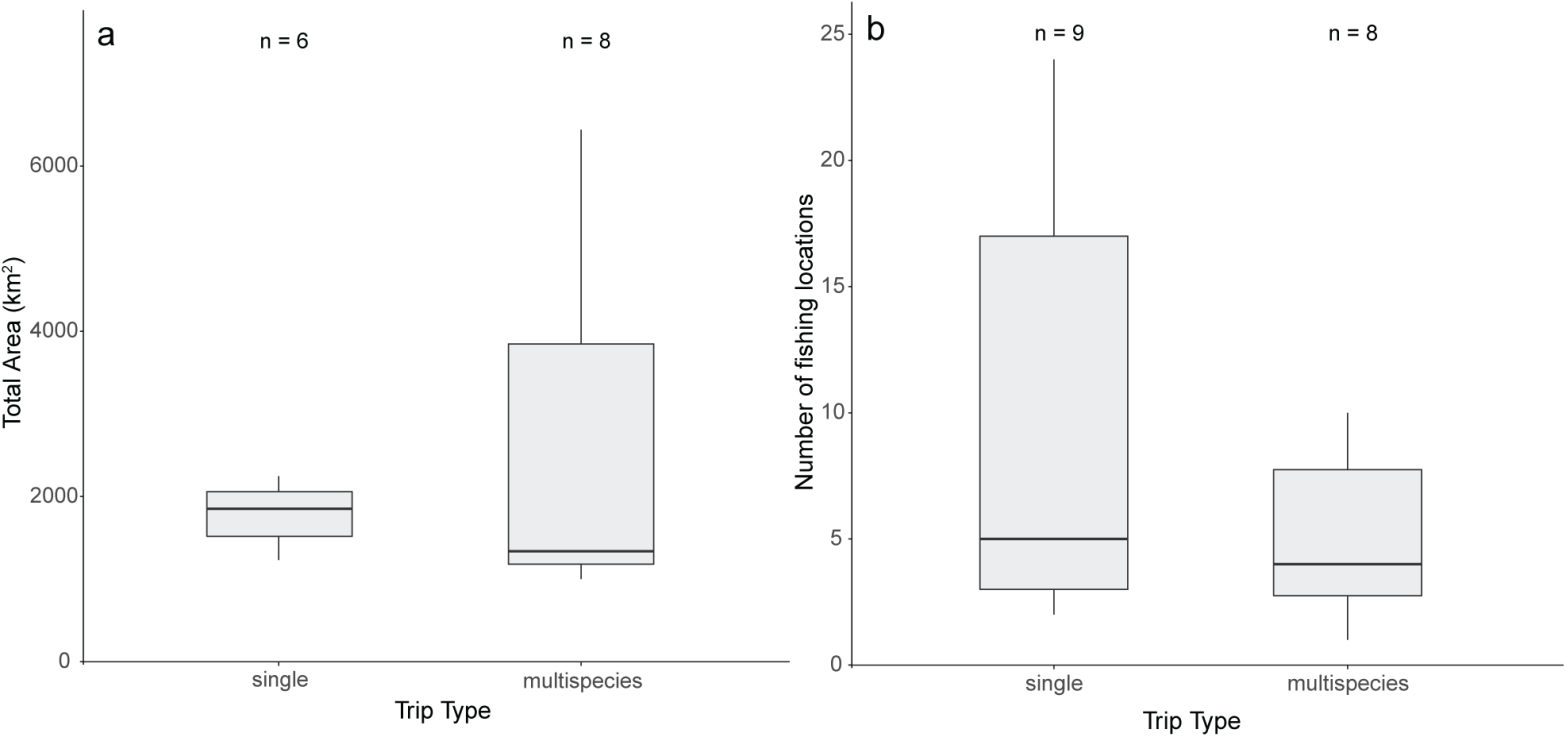
**Box and whisker plot displaying total fishing area (km**^**2**^**) (a) and the number of fishing locations (b) for Pacific halibut in Homer, categorized by whether the respondent targets single or multispecies trips for the majority of their charter fishing experience.** For both plots, the lower whisker extends from the first quartile to the lowest value within 1.5*IQR of the first quartile. The upper whisker extends from the third quartile to the highest value within 1.5*IQR of the third quartile. Outliers ranging above and below 1.5*IQR have been removed.

**Fig 7:**
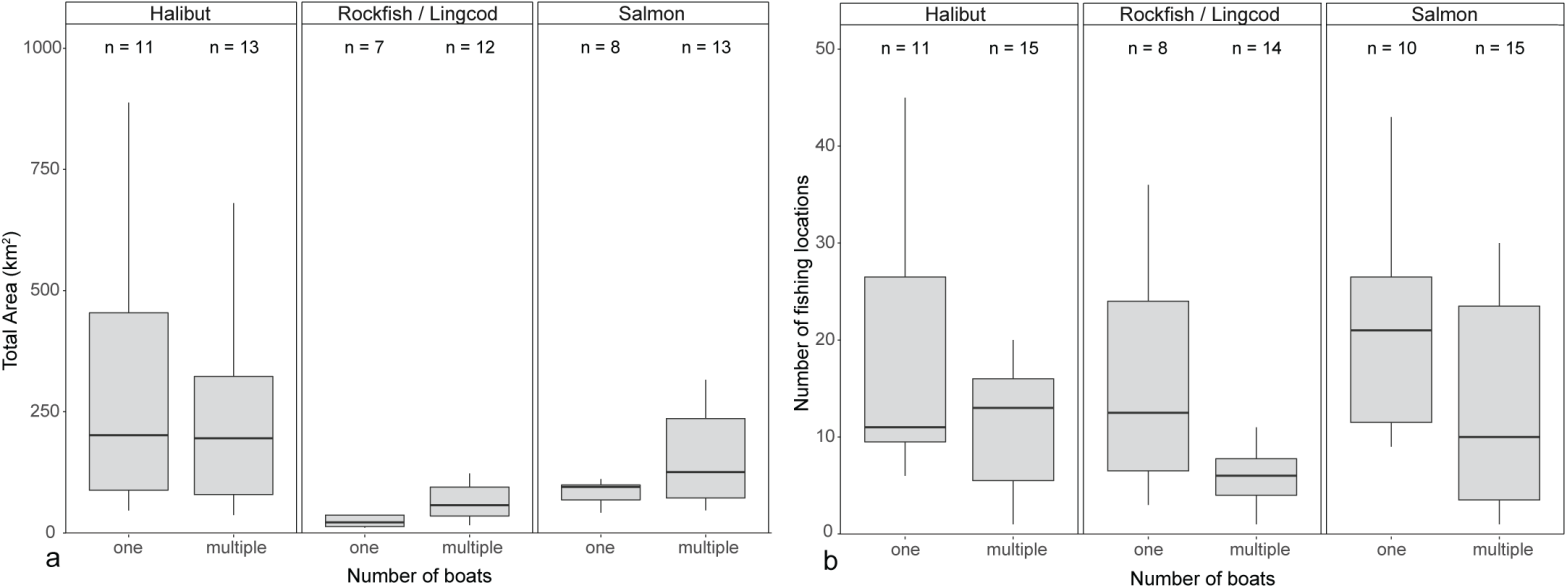
**Box and whisker plot displaying total fishing area (km**^**2**^**) (a) and the number of fishing locations (b) for Pacific halibut, lingcod/rockfish, and salmon fishing locations in Sitka, categorized by whether the respondent’s business had one or multiple charter boats.** For both plots, the lower whisker extends from the first quartile to the lowest value within 1.5*IQR of the first quartile. The upper whisker extends from the third quartile to the highest value within 1.5*IQR of the third quartile. Outliers ranging above and below 1.5*IQR have been removed.

Due to low sample sizes, we were unable to statistically compare the distributions of area fished and number of fishing locations among charter trip types. Qualitatively, the median area fished for single species trips in Homer was greater than for multispecies trips; however, the distribution of areas fished among respondents was wider for multispecies trips compared to single-species trips (Fig 6). The median area fished for rockfish/lingcod and Pacific salmon in Sitka was higher and the distribution of areas fished wider for multiple vessel businesses compared to single vessel businesses (Fig 7). For Pacific halibut, median area fished was similar between single vessel and multiple vessel businesses, with single vessel businesses showing a wider distribution of areas (Fig 7).

Reasons reported for spatial changes in fishing for Pacific halibut varied between Homer and Sitka. In Homer, the most frequently cited reasons for changes in the location and/or extent of fishing were changes in trip type (45%) and the price of fuel (45%; Table 5). In contrast, for Sitka, the most frequently cited reasons for spatial shifts were changes to Pacific halibut regulations (57%), and gaining experience or exploring new locations (38%; Table 5). For both locations, changes to Pacific halibut abundance or distribution were the third most frequently cited reason for spatial change (36% in Homer, 24% in Sitka; Table 5).

**Table 5:** Summary of the drivers of change to charter fishing locations targeting Pacific halibut.

Driver of change	Homer	Sitka
Change in trip type	45%	0%
Price of fuel	45%	0%
Changes to Pacific halibut abundance or distribution	36%	24%
Gaining experience/exploring new locations	27%	38%
Technological changes (e.g., fishing gear, boat, GPS)	18%	19%
Competitive pressure to target bigger/better halibut	18%	0%
Changes to Pacific halibut regulations	9%	57%
Change in captain’s fishing preferences	9%	0%
Changes to Pacific salmon regulations	0%	5%

## Discussion

Overall, the spatial footprint of charter fishing differed between Homer and Sitka respondents, with Homer operators consistently using larger areas for Pacific halibut than Sitka operators. For both Sitka and Homer respondents, the range of areas fished was greater for Pacific halibut than for rockfish/lingcod or Pacific salmon. Spatial patterns were qualitatively different between businesses specializing in single species trips and those that operated multispecies trips in Homer. Similarly, spatial patterns were qualitatively different between businesses with one vessel than those with multiple vessels in Sitka. Homer and Sitka showed opposite trends in core fishing location (CFL) area over time, with an overall decrease in Homer and an overall increase in Sitka.

Over the past twenty years, angler effort for charter bottomfish has changed from being focused primarily in Southcentral to a more even distribution between Southeast and Southcentral regions. In 1998, there were 96,158 angler-days of effort for charter bottomfish in Southcentral Alaska and 65,390 angler-days in Southeast [34]. By 2014, Southcentral saltwater charter bottomfish angler effort had increased to 109,981 angler-days, with a similar level of effort in Southeast at 100,940 angler-days [20]. Our results show that even as charter bottomfish effort has equalized between Southeast and Southcentral regions, median area fished for Pacific halibut has been consistently higher for charter operators in Homer compared to those in Sitka over the past twenty years (Fig 4).

Collectively, respondents’ fishing maps suggest that over the past twenty years, space use has been consistently different between Homer and Sitka charter boats, with Homer operators using larger fishing areas than Sitka operators to target all three species groups (Fig 4). The distributions of total area fished and the number of fishing locations for Pacific halibut were statistically different between Homer and Sitka respondents (Table 3). Space use between the two communities may differ, in part, due to the habitat and distribution of targeted charter species. During interviews, respondents in Homer explained that charter fishing locations for Pacific halibut and Pacific salmon can be found within several kilometers of town (Fig 2, S1 Fig), but lingcod locations are limited to sites greater than 20 km from Homer (S2 Fig). While charter fishing locations in Homer are naturally spatially segregated by target species, Sitka captains noted that there is suitable habitat for all three species groups within several kilometers of town (Fig 3, S3 Fig, S4 Fig).

In explaining why spatial changes occurred, Homer respondents identified the price of fuel as a major factor in changes to their spatial footprint (Table 5). From 2000–2005, the average price of #2 marine diesel in the port of Homer, Alaska, was $1.47 per gallon and by 2011–2015, it had increased to $3.39 per gallon (Fisheries Economics Data Program, Pacific States Marine Fisheries Commission, http://www.psmfc.org/efin/data/fuel.html). In the 2000s, Homer respondents had a median total fishing area for Pacific halibut that was 10 times greater than Sitka respondents (Fig 4); therefore, it is likely that the increase in fuel prices had a greater effect on Homer respondents than on Sitka respondents. In the 2010s, the majority of Homer respondents retracted the area they used to target Pacific halibut to reduce fuel consumption and, therefore, cost (Table 3 and Fig 7). Two Homer charter operators explained the role that fuel costs played in their decision to stop fishing further south:

“*Back in the 80s*, *I probably fished there 30*, *40% of the time*. *I used to take my big boat down there*. *One day I went down there and it cost $700*. *I didn’t go there for a while after that*. *There was a time when you’d see 20 or 30 boats down there and now very few boats go down there*.”–Anonymous #1

“*We used to fish around the Barren Islands a lot*, *the fleet did*, *when I was first here in the 90s*. *We used to go down there on a regular basis*. *The fuel prices affected that because if fuel prices get outrageously high*, *your rates will be outrageously high for a lot of people* [customers] *too*. *That created a disincentive to go down there*.”–Anonymous #2

The importance of fuel costs in determining charter fishing behavior has been found in other studies as well. Research on the Ohio Lake Erie charter industry showed that in 2006, for boat-owning captains, boat fuel was the largest (29%) annual operating expense [35]. Additionally, the cost of fuel was the most cited concern facing the charter industry in Lake Erie (64% of 232 respondents) [35]. In an analysis of Cook Inlet saltwater sportfishing charter operations near Homer, Alaska, fuel was the second greatest expenditure, after proprietor income [36], highlighting the potential impact of fuel costs on charter businesses in Homer.

In Sitka, the price of fuel was not cited by any respondent as affecting fishing locations or area fished. Rather, Sitka respondents most frequently cited Pacific halibut regulations as the driver of spatial change (57%; Table 5). A Local Area Management Plan (LAMP) was implemented in 1999 in which Pacific halibut charter fishing was no longer permitted inside Sitka Sound in the summer months [27]. In addition to LAMP, which pushed charter Pacific halibut fishing to locations greater than 30 km from town (Fig 3), starting in the late 2000s, the Sitka charter fleet faced almost yearly regulatory changes regarding Pacific halibut, especially to bag and size limits (Table 1). In Homer, where regulations have been more stable and less restrictive, just 9% of respondents indicated that regulations had affected their fishing locations. Similarly, in a recreational fishery in Washington State, only 5% of respondents cited the role of regulations in affecting their fishing locations [31]. These differences in major drivers of spatial change highlight that Homer respondents may be more vulnerable to economic variables such as fuel prices, while Sitka respondents have been more affected by fisheries regulations.

The charter industry operates under different business models in Homer and Sitka, which may explain some of the differences in spatial attributes of fishing among respondents in those communities. Homer has charter businesses that specialize in targeting Pacific halibut and those that target multiple species. Respondents who mainly operated multispecies trips had a wider distribution, but smaller median area for Pacific halibut than those operating primarily single species trips, highlighting that the type of trips offered at a charter business likely affects fishing patterns; however, the spatial attributes could not be statistically compared between these respondent groups due to low sample sizes (Fig 5). In Homer, trip type was among the most frequently cited reasons for spatial change (Table 5), showing that trip characteristics, such as the targeted species and trip duration, can be indicators of where fishing occurs. Because of reliable Pacific salmon fishing, Sitka charter businesses operate primarily multispecies trips, most often consisting of Pacific halibut and Pacific salmon [20]. In Sitka, both the total area used and the number of discrete locations differed among respondents based on the size of their charter business (Fig 7). For Pacific halibut, multiple-boat businesses used a greater number of individual locations than single boat businesses (Fig 7). For rockfish/lingcod and Pacific salmon, single boat operators had greater median number of individual locations, but used smaller total areas. Again, while they could not be evaluated statistically, these qualitative differences between single- and multiple-boat businesses highlight that fishing behavior of charter operators may differ based on the size of the business (Fig 7).

People operate a diversity of business sizes in the saltwater sportfishing charter industry in Alaska, from owner-operator vessels to fishing lodges with multiple boats. Additionally, charter operators can specialize in single species trips or pursue multiple species based on their skills, customer base, and marketing strategy. The diversity of charter fishing strategies in Alaska allows these groups to respond differently to social, environmental, and regulatory perturbations. Response diversity has traditionally been defined as the range of responses of species within a functional group to environmental change, particularly in the context of maintaining ecosystem function [37]. In recent years, response diversity has expanded to research on social-ecological systems and can be defined as the range of human reactions to the same challenges, opportunities, or risks [38]. Social-ecological resilience has been attributed to response diversity, which may be a crucial part of the adaptive capacity of a system [37, 38]. In Alaska, variation in business sizes and trip types may allow charter fishing to persist as a viable industry long term. For example, in 2014, a regulation was implemented in Southcentral Alaska that prohibited charter operators from fishing for Pacific halibut on Thursdays (Table 1). For businesses that only targeted Pacific halibut, the Thursday closure forced them to either reduce business operations or quickly find an alternative target species; however, for businesses that specialized in multiple species, the Thursday closure removed Pacific halibut from the repertoire on that day, but operators were still able to pursue other species (e.g., lingcod and Pacific salmon). Undoubtedly, the day closure reduced the flexibility of charter businesses by placing additional constraints on them, but it affected various actors differently. We argue that the resilience of the charter industry in part depends on the diversity of business strategies within the industry and variation in how different individuals respond to change.

In Alaska, diversity in charter fishing originates from endogenous factors within charter businesses (e.g., variation in business models), but also from exogenous drivers such as differences in regulatory restrictions between regions. In Sitka, with the bag limit already at one fish, there have been yearly changes to Pacific halibut size limits for the past five years (Table 1). In Homer, few limits were placed on charter fishing until 2014, but the community has faced a faster pace of change since then, with three new regulations simultaneously added in 2015 (Table 1). The constancy of incremental regulatory changes may give Sitka businesses the outlook stability needed pursue long-term business goals, such as marketing reliable trips to customers or expanding the business, rather than having to quickly adapt to sudden large changes. Barring major future changes, we might expect stability in the spatial footprint of charter fishing in Sitka. However, the uncertain climate of future regulatory change in Homer makes it difficult to predict how charter operators will shift their behavior, including target species and locations, to accommodate new regulations. These differences between Homer and Sitka illustrate the importance of recognizing the place-based context in which policies for bag limits, spatial closures, or other management changes are made so that the impacts on local people, and associated ecological impacts, can be appropriately assessed.

## Supporting information

S1 Fig Salmon fishing locations in Homer for 1990–1999, 2000–2004, 2005–2009, and 2010–2015. Locations are displayed by the percentage of respondents who fished during that time period.
(TIF)

S2 Fig Lingcod and rockfish fishing locations in Homer for 1990–1999, 2000–2004, 2005–2009, and 2010–2015. Locations are displayed by the percentage of respondents who fished during that time period.
(TIF)

S3 Fig Lingcod and rockfish fishing locations in Sitka for 2000–2004, 2005–2009, and 2010–2015. Locations for 1990s are not shown due to low sample size (<5 respondents). Locations are displayed by the percentage of respondents who fished during that time period.
(TIF)

S4 Fig Salmon fishing locations in Sitka for 1990–1999, 2000–2004, 2005–2009, and 2010–2015. Locations are displayed by the percentage of respondents who fished during that time period.
(TIF)

S1 Dataset Total fishing area (km^2^) and the number of fishing locations for Pacific halibut, rockfish/lingcod, and salmon for the 1990s, 2000s, and 2010s in Homer and Sitka. Data include: respondent identification number (ID), species group (Species), decade in which the respondent fished (Decade), total area (TotalArea.sq.km), number of fishing locations (NumberLocations), and region.
(XLSX)

S2 Dataset Total fishing area (km^2^) and the number of fishing locations for Pacific halibut in Homer, categorized by whether the respondent targets single or multispecies trips for the majority of their charter fishing experience. Data include: respondent identification number (ID), total area (TotalArea.sq.km), number of fishing locations (NumberLocations), and whether the respondent targets single or multispecies trips (TripType).
(XLSX)

S3 Dataset Total fishing area (km^2^) and the number of fishing locations for Pacific halibut, lingcod/rockfish, and salmon fishing locations in Sitka, categorized by whether the respondent’s business had one or multiple charter boats. Data include: respondent identification number (ID), species group (Species), total area (TotalArea.sq.km), and number of fishing locations (NumberLocations).
(XLSX)

S1 File A folder containing files of Pacific halibut fishing locations in Homer and Sitka for 1990–1999, 2000–2004, 2005–2009, and 2010–2015. Files are in TIFF format to be displayed in ESRI ArcGIS 10.2 or higher. The attribute table for each TIFF file contains a column (PERCENT_RE) identifying the percentage of respondents in that time period fishing that 1.5 x 1.5 km grid cell (i.e., Figs 2 and 3). All files are projected in Alaska Albers coordinate system (NAD 1983–2011 Alaska Albers, WKID: 102966, Authority: ESRI). ADF&G statistical fishing areas are not provided in this dataset because the authority to share that data rests with ADF&G.
(ZIP)

S2 File A folder containing files of salmon fishing locations in Homer and Sitka for 1990–1999, 2000–2004, 2005–2009, and 2010–2015. Files are in TIFF format to be displayed in ESRI ArcGIS 10.2 or higher. The attribute table for each TIFF file contains a column (PERCENT_RE) identifying the percentage of respondents in that time period fishing that 1.5 x 1.5 km grid cell (i.e., S1 Fig, S4 Fig). All files are projected in Alaska Albers coordinate system (NAD 1983–2011 Alaska Albers, WKID: 102966, Authority: ESRI).
(ZIP)

S3 File A folder containing files of lingcod and rockfish fishing locations in Homer and Sitka for 1990–1999, 2000–2004, 2005–2009, and 2010–2015. Files are in TIFF format to be displayed in ESRI ArcGIS 10.2 or higher. The attribute table for each TIFF file contains a column (PERCENT_RE) identifying the percentage of respondents in that time period fishing that 1.5 x 1.5 km grid cell (i.e., S2 Fig, S3 Fig). All files are projected in Alaska Albers coordinate system (NAD 1983–2011 Alaska Albers, WKID: 102966, Authority: ESRI).
(ZIP)

S1 Text Questions administered to interview respondents. After acquiring informed consent, the interview team asked a series of questions to the participant on charter fishing experience, charter business information, and fishing locations.
(DOCX)
