# Supplementary figures and images for: Evaluating patterns and drivers of spatial change in the recreational guided fishing sector in Alaska
Source: PLoS One. 2017 Jun 20;12(6):e0179584. doi: 10.1371/journal.pone.0179584 (PMC5478146; doi:10.1371/journal.pone.0179584)

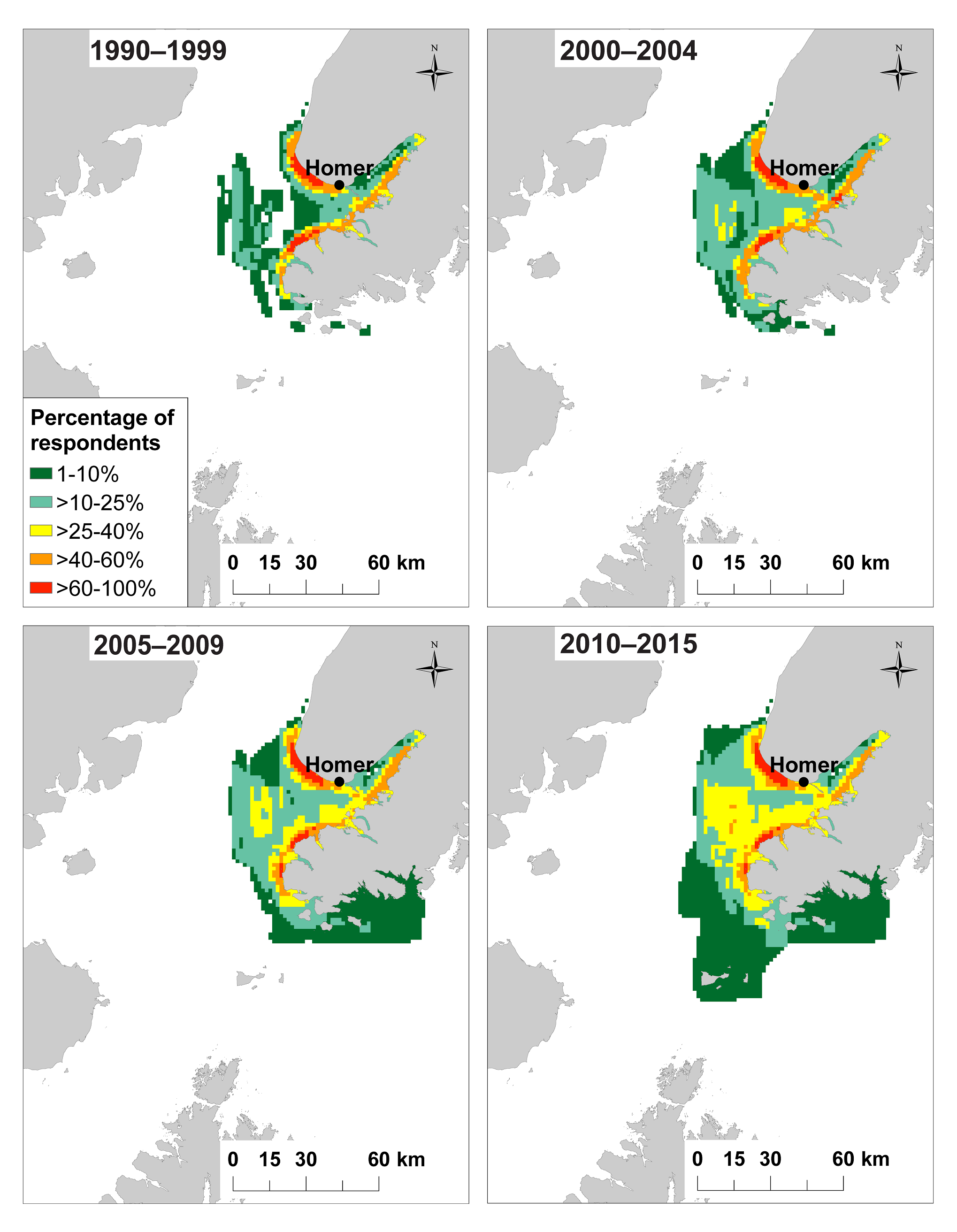

Supplement: S1 Fig — Locations are displayed by the percentage of respondents who fished during that time period. (TIF) [file pone.0179584.s001.tif]

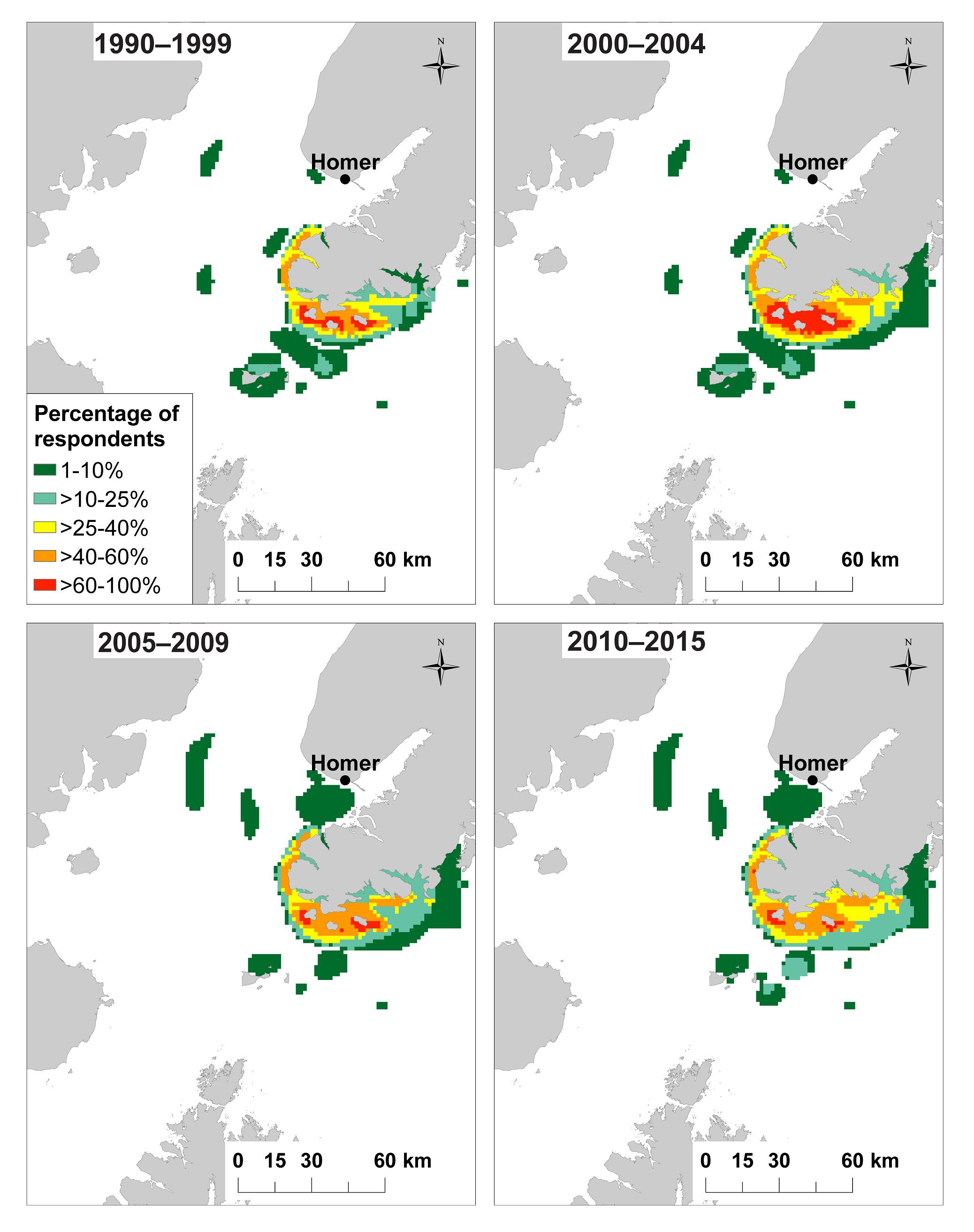

Supplement: S2 Fig — Locations are displayed by the percentage of respondents who fished during that time period. (TIF) [file pone.0179584.s002.tif]

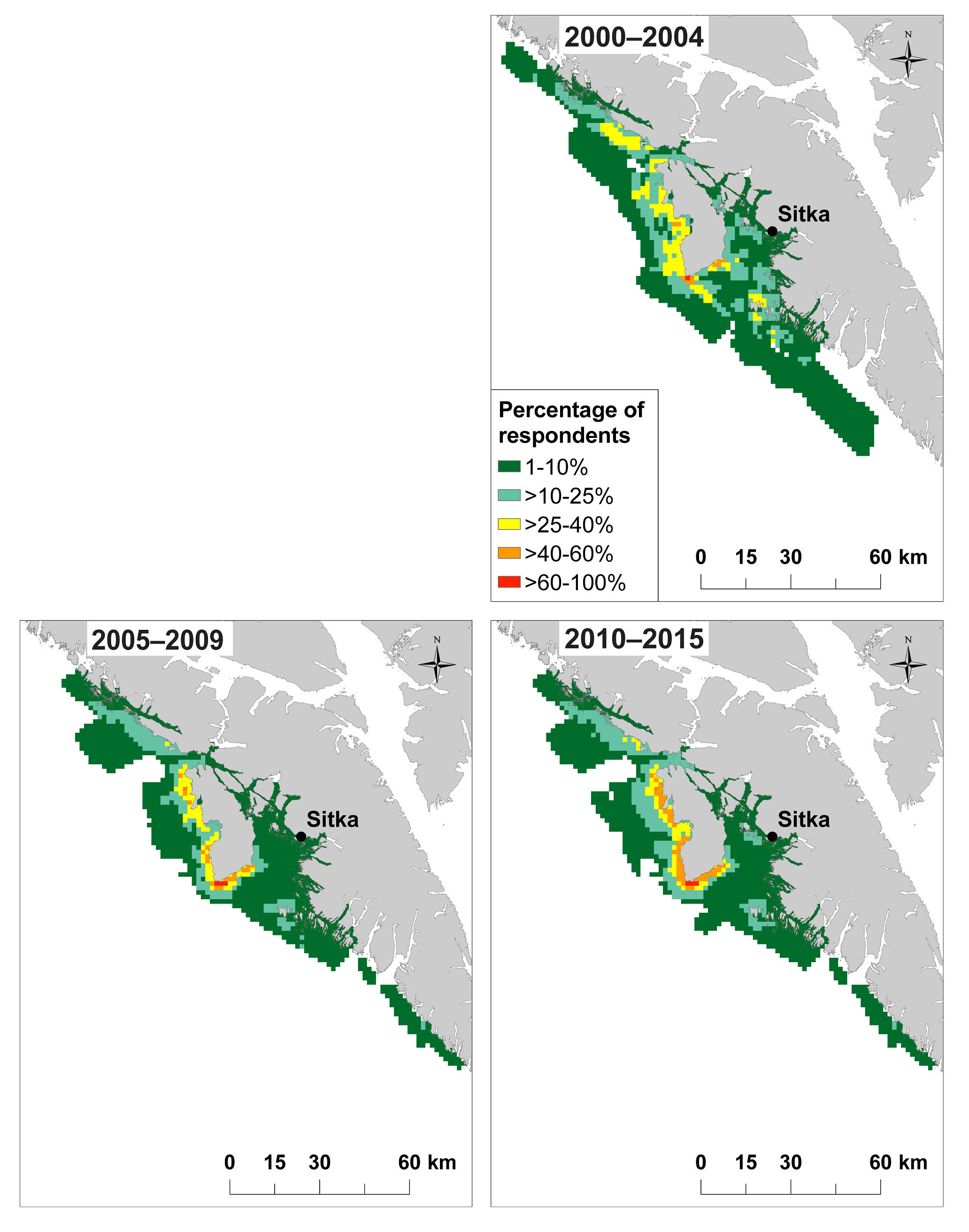

Supplement: S3 Fig — Locations for 1990s are not shown due to low sample size (<5 respondents). Locations are displayed by the percentage of respondents who fished during that time period. (TIF) [file pone.0179584.s003.tif]

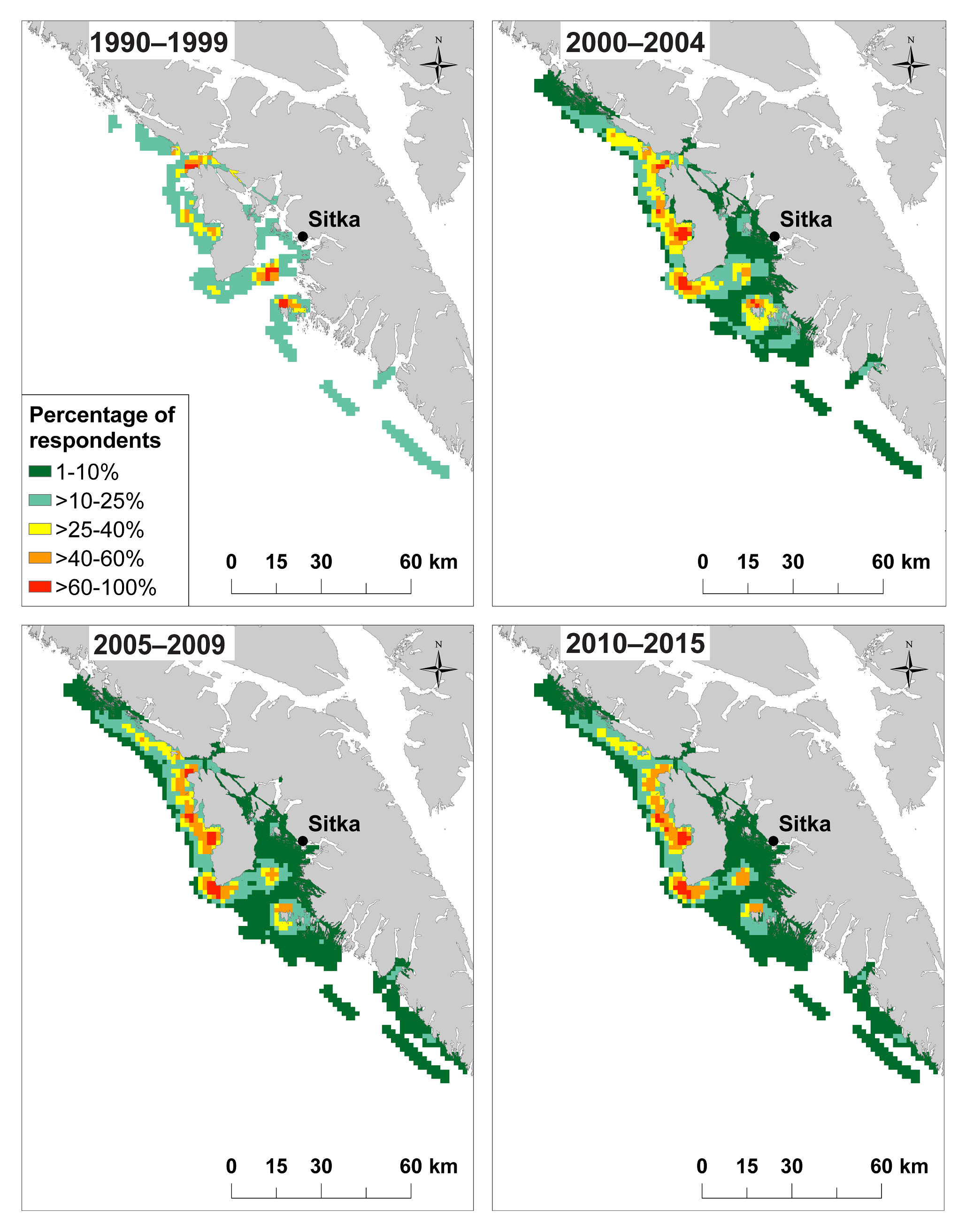

Supplement: S4 Fig — Locations are displayed by the percentage of respondents who fished during that time period. (TIF) [file pone.0179584.s004.tif]

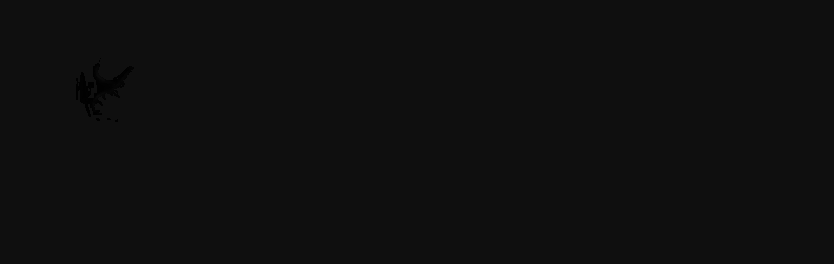

Supplement: S2 File — Files are in TIFF format to be displayed in ESRI ArcGIS 10.2 or higher. The attribute table for each TIFF file contains a column (PERCENT_RE) identifying the percentage of respondents in that time period fishing that 1.5 x 1.5 km grid cell (i.e., S1 Fig, S4 Fig). All files are projected in Alaska Albers coordinate system (NAD 1983–2011 Alaska Albers, WKID: 102966, Authority: ESRI). (ZIP) [file pone.0179584.s009.zip › S9_salmon/Homer_salmon_1990s.tif]

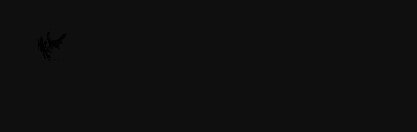

Supplement: S2 File — Files are in TIFF format to be displayed in ESRI ArcGIS 10.2 or higher. The attribute table for each TIFF file contains a column (PERCENT_RE) identifying the percentage of respondents in that time period fishing that 1.5 x 1.5 km grid cell (i.e., S1 Fig, S4 Fig). All files are projected in Alaska Albers coordinate system (NAD 1983–2011 Alaska Albers, WKID: 102966, Authority: ESRI). (ZIP) [file pone.0179584.s009.zip › S9_salmon/Homer_salmon_1990s.tif.ovr]

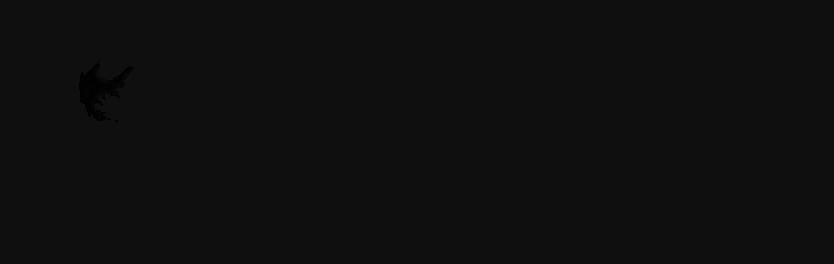

Supplement: S2 File — Files are in TIFF format to be displayed in ESRI ArcGIS 10.2 or higher. The attribute table for each TIFF file contains a column (PERCENT_RE) identifying the percentage of respondents in that time period fishing that 1.5 x 1.5 km grid cell (i.e., S1 Fig, S4 Fig). All files are projected in Alaska Albers coordinate system (NAD 1983–2011 Alaska Albers, WKID: 102966, Authority: ESRI). (ZIP) [file pone.0179584.s009.zip › S9_salmon/Homer_salmon_2000_2004.tif]

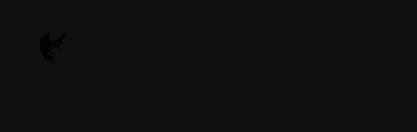

Supplement: S2 File — Files are in TIFF format to be displayed in ESRI ArcGIS 10.2 or higher. The attribute table for each TIFF file contains a column (PERCENT_RE) identifying the percentage of respondents in that time period fishing that 1.5 x 1.5 km grid cell (i.e., S1 Fig, S4 Fig). All files are projected in Alaska Albers coordinate system (NAD 1983–2011 Alaska Albers, WKID: 102966, Authority: ESRI). (ZIP) [file pone.0179584.s009.zip › S9_salmon/Homer_salmon_2000_2004.tif.ovr]

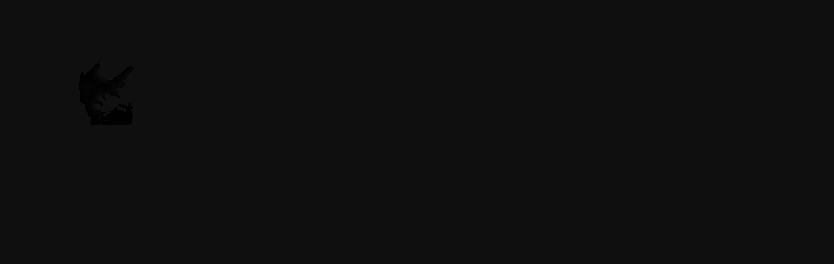

Supplement: S2 File — Files are in TIFF format to be displayed in ESRI ArcGIS 10.2 or higher. The attribute table for each TIFF file contains a column (PERCENT_RE) identifying the percentage of respondents in that time period fishing that 1.5 x 1.5 km grid cell (i.e., S1 Fig, S4 Fig). All files are projected in Alaska Albers coordinate system (NAD 1983–2011 Alaska Albers, WKID: 102966, Authority: ESRI). (ZIP) [file pone.0179584.s009.zip › S9_salmon/Homer_salmon_2005_2009.tif]

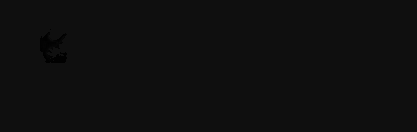

Supplement: S2 File — Files are in TIFF format to be displayed in ESRI ArcGIS 10.2 or higher. The attribute table for each TIFF file contains a column (PERCENT_RE) identifying the percentage of respondents in that time period fishing that 1.5 x 1.5 km grid cell (i.e., S1 Fig, S4 Fig). All files are projected in Alaska Albers coordinate system (NAD 1983–2011 Alaska Albers, WKID: 102966, Authority: ESRI). (ZIP) [file pone.0179584.s009.zip › S9_salmon/Homer_salmon_2005_2009.tif.ovr]

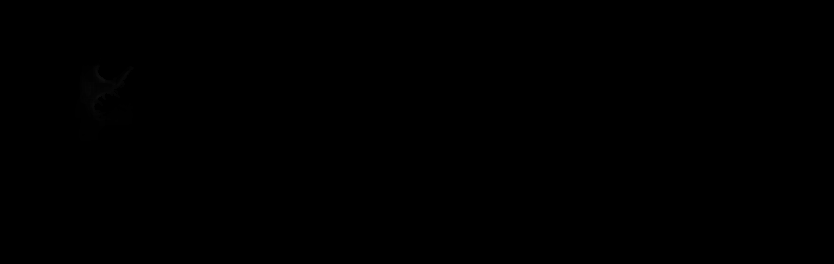

Supplement: S2 File — Files are in TIFF format to be displayed in ESRI ArcGIS 10.2 or higher. The attribute table for each TIFF file contains a column (PERCENT_RE) identifying the percentage of respondents in that time period fishing that 1.5 x 1.5 km grid cell (i.e., S1 Fig, S4 Fig). All files are projected in Alaska Albers coordinate system (NAD 1983–2011 Alaska Albers, WKID: 102966, Authority: ESRI). (ZIP) [file pone.0179584.s009.zip › S9_salmon/Homer_salmon_2010_2015.tif]

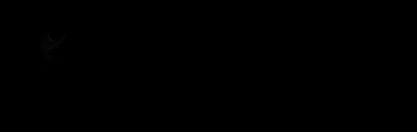

Supplement: S2 File — Files are in TIFF format to be displayed in ESRI ArcGIS 10.2 or higher. The attribute table for each TIFF file contains a column (PERCENT_RE) identifying the percentage of respondents in that time period fishing that 1.5 x 1.5 km grid cell (i.e., S1 Fig, S4 Fig). All files are projected in Alaska Albers coordinate system (NAD 1983–2011 Alaska Albers, WKID: 102966, Authority: ESRI). (ZIP) [file pone.0179584.s009.zip › S9_salmon/Homer_salmon_2010_2015.tif.ovr]

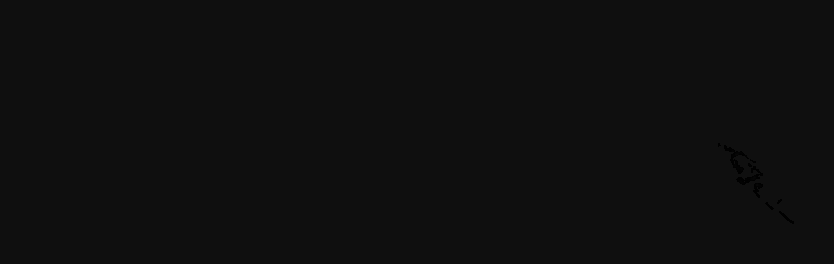

Supplement: S2 File — Files are in TIFF format to be displayed in ESRI ArcGIS 10.2 or higher. The attribute table for each TIFF file contains a column (PERCENT_RE) identifying the percentage of respondents in that time period fishing that 1.5 x 1.5 km grid cell (i.e., S1 Fig, S4 Fig). All files are projected in Alaska Albers coordinate system (NAD 1983–2011 Alaska Albers, WKID: 102966, Authority: ESRI). (ZIP) [file pone.0179584.s009.zip › S9_salmon/Sitka_salmon_1990s.tif]

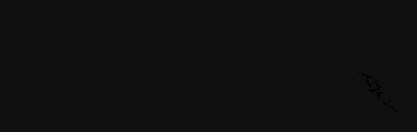

Supplement: S2 File — Files are in TIFF format to be displayed in ESRI ArcGIS 10.2 or higher. The attribute table for each TIFF file contains a column (PERCENT_RE) identifying the percentage of respondents in that time period fishing that 1.5 x 1.5 km grid cell (i.e., S1 Fig, S4 Fig). All files are projected in Alaska Albers coordinate system (NAD 1983–2011 Alaska Albers, WKID: 102966, Authority: ESRI). (ZIP) [file pone.0179584.s009.zip › S9_salmon/Sitka_salmon_1990s.tif.ovr]

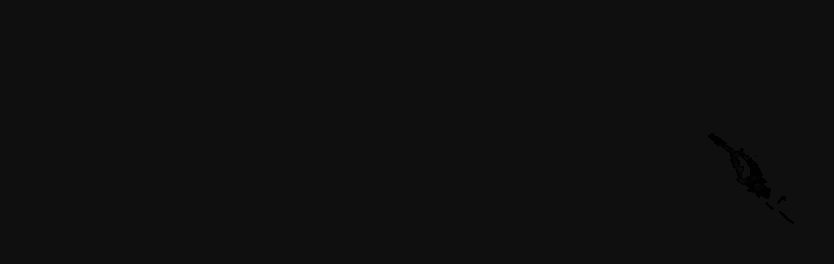

Supplement: S2 File — Files are in TIFF format to be displayed in ESRI ArcGIS 10.2 or higher. The attribute table for each TIFF file contains a column (PERCENT_RE) identifying the percentage of respondents in that time period fishing that 1.5 x 1.5 km grid cell (i.e., S1 Fig, S4 Fig). All files are projected in Alaska Albers coordinate system (NAD 1983–2011 Alaska Albers, WKID: 102966, Authority: ESRI). (ZIP) [file pone.0179584.s009.zip › S9_salmon/Sitka_salmon_2000_2004.tif]

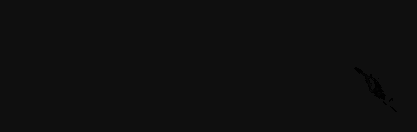

Supplement: S2 File — Files are in TIFF format to be displayed in ESRI ArcGIS 10.2 or higher. The attribute table for each TIFF file contains a column (PERCENT_RE) identifying the percentage of respondents in that time period fishing that 1.5 x 1.5 km grid cell (i.e., S1 Fig, S4 Fig). All files are projected in Alaska Albers coordinate system (NAD 1983–2011 Alaska Albers, WKID: 102966, Authority: ESRI). (ZIP) [file pone.0179584.s009.zip › S9_salmon/Sitka_salmon_2000_2004.tif.ovr]

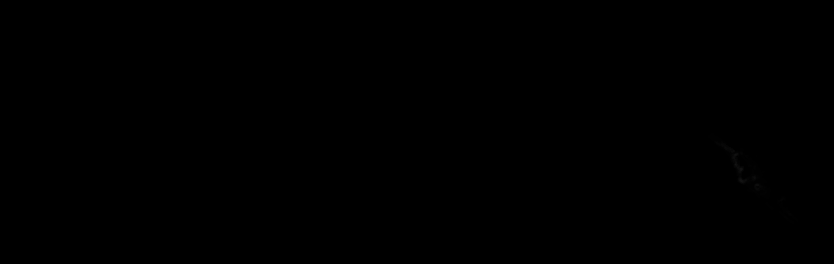

Supplement: S2 File — Files are in TIFF format to be displayed in ESRI ArcGIS 10.2 or higher. The attribute table for each TIFF file contains a column (PERCENT_RE) identifying the percentage of respondents in that time period fishing that 1.5 x 1.5 km grid cell (i.e., S1 Fig, S4 Fig). All files are projected in Alaska Albers coordinate system (NAD 1983–2011 Alaska Albers, WKID: 102966, Authority: ESRI). (ZIP) [file pone.0179584.s009.zip › S9_salmon/Sitka_salmon_2005_2009.tif]

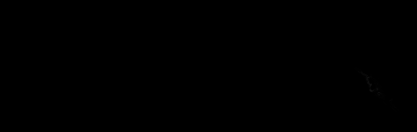

Supplement: S2 File — Files are in TIFF format to be displayed in ESRI ArcGIS 10.2 or higher. The attribute table for each TIFF file contains a column (PERCENT_RE) identifying the percentage of respondents in that time period fishing that 1.5 x 1.5 km grid cell (i.e., S1 Fig, S4 Fig). All files are projected in Alaska Albers coordinate system (NAD 1983–2011 Alaska Albers, WKID: 102966, Authority: ESRI). (ZIP) [file pone.0179584.s009.zip › S9_salmon/Sitka_salmon_2005_2009.tif.ovr]

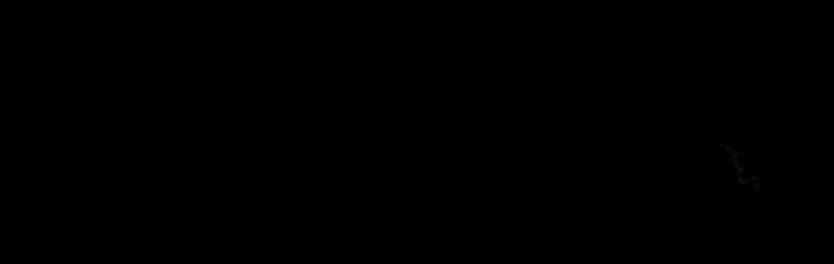

Supplement: S2 File — Files are in TIFF format to be displayed in ESRI ArcGIS 10.2 or higher. The attribute table for each TIFF file contains a column (PERCENT_RE) identifying the percentage of respondents in that time period fishing that 1.5 x 1.5 km grid cell (i.e., S1 Fig, S4 Fig). All files are projected in Alaska Albers coordinate system (NAD 1983–2011 Alaska Albers, WKID: 102966, Authority: ESRI). (ZIP) [file pone.0179584.s009.zip › S9_salmon/Sitka_salmon_2010_2015.tif]

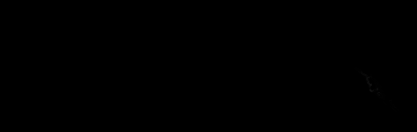

Supplement: S2 File — Files are in TIFF format to be displayed in ESRI ArcGIS 10.2 or higher. The attribute table for each TIFF file contains a column (PERCENT_RE) identifying the percentage of respondents in that time period fishing that 1.5 x 1.5 km grid cell (i.e., S1 Fig, S4 Fig). All files are projected in Alaska Albers coordinate system (NAD 1983–2011 Alaska Albers, WKID: 102966, Authority: ESRI). (ZIP) [file pone.0179584.s009.zip › S9_salmon/Sitka_salmon_2010_2015.tif.ovr]

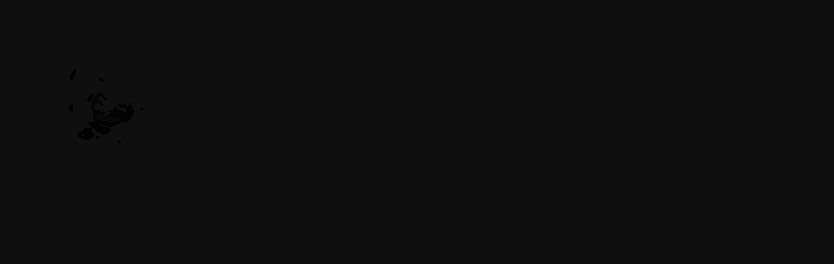

Supplement: S3 File — Files are in TIFF format to be displayed in ESRI ArcGIS 10.2 or higher. The attribute table for each TIFF file contains a column (PERCENT_RE) identifying the percentage of respondents in that time period fishing that 1.5 x 1.5 km grid cell (i.e., S2 Fig, S3 Fig). All files are projected in Alaska Albers coordinate system (NAD 1983–2011 Alaska Albers, WKID: 102966, Authority: ESRI). (ZIP) [file pone.0179584.s010.zip › S10_lingcod_rockfish/Homer_lingcod_rockfish_1990s.tif]

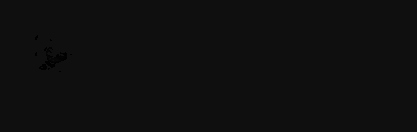

Supplement: S3 File — Files are in TIFF format to be displayed in ESRI ArcGIS 10.2 or higher. The attribute table for each TIFF file contains a column (PERCENT_RE) identifying the percentage of respondents in that time period fishing that 1.5 x 1.5 km grid cell (i.e., S2 Fig, S3 Fig). All files are projected in Alaska Albers coordinate system (NAD 1983–2011 Alaska Albers, WKID: 102966, Authority: ESRI). (ZIP) [file pone.0179584.s010.zip › S10_lingcod_rockfish/Homer_lingcod_rockfish_1990s.tif.ovr]

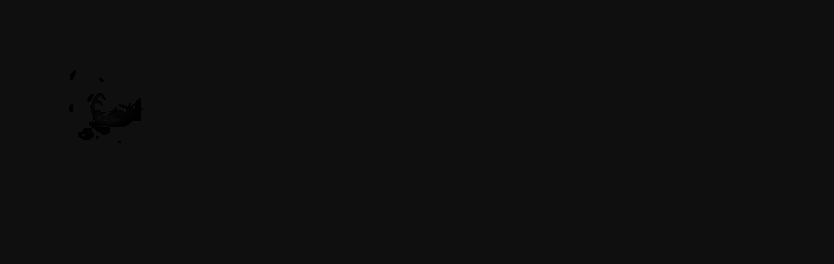

Supplement: S3 File — Files are in TIFF format to be displayed in ESRI ArcGIS 10.2 or higher. The attribute table for each TIFF file contains a column (PERCENT_RE) identifying the percentage of respondents in that time period fishing that 1.5 x 1.5 km grid cell (i.e., S2 Fig, S3 Fig). All files are projected in Alaska Albers coordinate system (NAD 1983–2011 Alaska Albers, WKID: 102966, Authority: ESRI). (ZIP) [file pone.0179584.s010.zip › S10_lingcod_rockfish/Homer_lingcod_rockfish_2000_2004.tif]

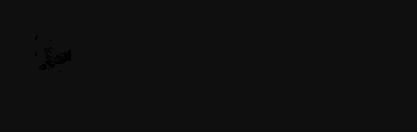

Supplement: S3 File — Files are in TIFF format to be displayed in ESRI ArcGIS 10.2 or higher. The attribute table for each TIFF file contains a column (PERCENT_RE) identifying the percentage of respondents in that time period fishing that 1.5 x 1.5 km grid cell (i.e., S2 Fig, S3 Fig). All files are projected in Alaska Albers coordinate system (NAD 1983–2011 Alaska Albers, WKID: 102966, Authority: ESRI). (ZIP) [file pone.0179584.s010.zip › S10_lingcod_rockfish/Homer_lingcod_rockfish_2000_2004.tif.ovr]

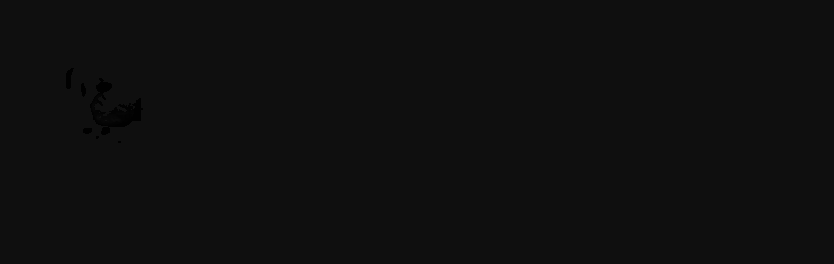

Supplement: S3 File — Files are in TIFF format to be displayed in ESRI ArcGIS 10.2 or higher. The attribute table for each TIFF file contains a column (PERCENT_RE) identifying the percentage of respondents in that time period fishing that 1.5 x 1.5 km grid cell (i.e., S2 Fig, S3 Fig). All files are projected in Alaska Albers coordinate system (NAD 1983–2011 Alaska Albers, WKID: 102966, Authority: ESRI). (ZIP) [file pone.0179584.s010.zip › S10_lingcod_rockfish/Homer_lingcod_rockfish_2005_2009.tif]

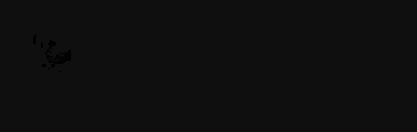

Supplement: S3 File — Files are in TIFF format to be displayed in ESRI ArcGIS 10.2 or higher. The attribute table for each TIFF file contains a column (PERCENT_RE) identifying the percentage of respondents in that time period fishing that 1.5 x 1.5 km grid cell (i.e., S2 Fig, S3 Fig). All files are projected in Alaska Albers coordinate system (NAD 1983–2011 Alaska Albers, WKID: 102966, Authority: ESRI). (ZIP) [file pone.0179584.s010.zip › S10_lingcod_rockfish/Homer_lingcod_rockfish_2005_2009.tif.ovr]

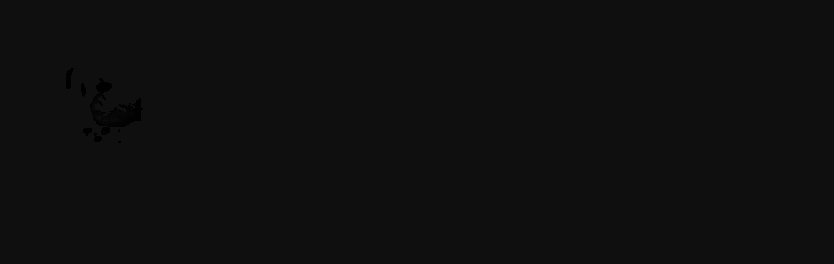

Supplement: S3 File — Files are in TIFF format to be displayed in ESRI ArcGIS 10.2 or higher. The attribute table for each TIFF file contains a column (PERCENT_RE) identifying the percentage of respondents in that time period fishing that 1.5 x 1.5 km grid cell (i.e., S2 Fig, S3 Fig). All files are projected in Alaska Albers coordinate system (NAD 1983–2011 Alaska Albers, WKID: 102966, Authority: ESRI). (ZIP) [file pone.0179584.s010.zip › S10_lingcod_rockfish/Homer_lingcod_rockfish_2010_2015.tif]

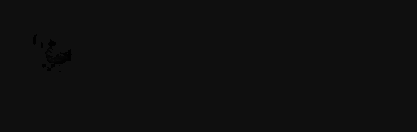

Supplement: S3 File — Files are in TIFF format to be displayed in ESRI ArcGIS 10.2 or higher. The attribute table for each TIFF file contains a column (PERCENT_RE) identifying the percentage of respondents in that time period fishing that 1.5 x 1.5 km grid cell (i.e., S2 Fig, S3 Fig). All files are projected in Alaska Albers coordinate system (NAD 1983–2011 Alaska Albers, WKID: 102966, Authority: ESRI). (ZIP) [file pone.0179584.s010.zip › S10_lingcod_rockfish/Homer_lingcod_rockfish_2010_2015.tif.ovr]

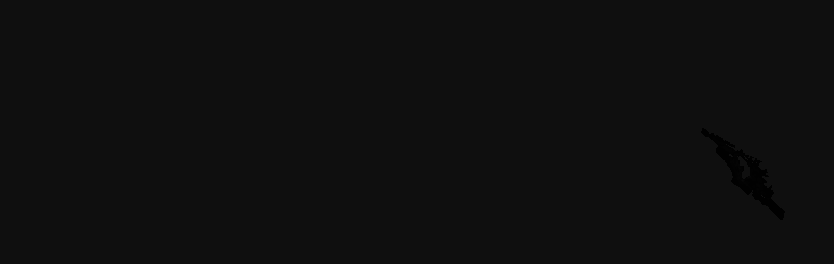

Supplement: S3 File — Files are in TIFF format to be displayed in ESRI ArcGIS 10.2 or higher. The attribute table for each TIFF file contains a column (PERCENT_RE) identifying the percentage of respondents in that time period fishing that 1.5 x 1.5 km grid cell (i.e., S2 Fig, S3 Fig). All files are projected in Alaska Albers coordinate system (NAD 1983–2011 Alaska Albers, WKID: 102966, Authority: ESRI). (ZIP) [file pone.0179584.s010.zip › S10_lingcod_rockfish/Sitka_lingcod_rockfish_2000_2004.tif]

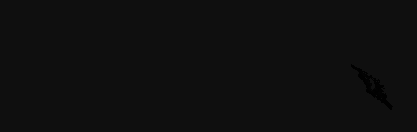

Supplement: S3 File — Files are in TIFF format to be displayed in ESRI ArcGIS 10.2 or higher. The attribute table for each TIFF file contains a column (PERCENT_RE) identifying the percentage of respondents in that time period fishing that 1.5 x 1.5 km grid cell (i.e., S2 Fig, S3 Fig). All files are projected in Alaska Albers coordinate system (NAD 1983–2011 Alaska Albers, WKID: 102966, Authority: ESRI). (ZIP) [file pone.0179584.s010.zip › S10_lingcod_rockfish/Sitka_lingcod_rockfish_2000_2004.tif.ovr]

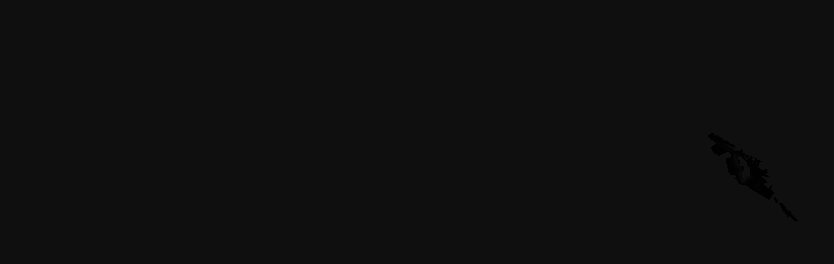

Supplement: S3 File — Files are in TIFF format to be displayed in ESRI ArcGIS 10.2 or higher. The attribute table for each TIFF file contains a column (PERCENT_RE) identifying the percentage of respondents in that time period fishing that 1.5 x 1.5 km grid cell (i.e., S2 Fig, S3 Fig). All files are projected in Alaska Albers coordinate system (NAD 1983–2011 Alaska Albers, WKID: 102966, Authority: ESRI). (ZIP) [file pone.0179584.s010.zip › S10_lingcod_rockfish/Sitka_lingcod_rockfish_2005_2009.tif]

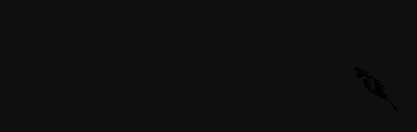

Supplement: S3 File — Files are in TIFF format to be displayed in ESRI ArcGIS 10.2 or higher. The attribute table for each TIFF file contains a column (PERCENT_RE) identifying the percentage of respondents in that time period fishing that 1.5 x 1.5 km grid cell (i.e., S2 Fig, S3 Fig). All files are projected in Alaska Albers coordinate system (NAD 1983–2011 Alaska Albers, WKID: 102966, Authority: ESRI). (ZIP) [file pone.0179584.s010.zip › S10_lingcod_rockfish/Sitka_lingcod_rockfish_2005_2009.tif.ovr]

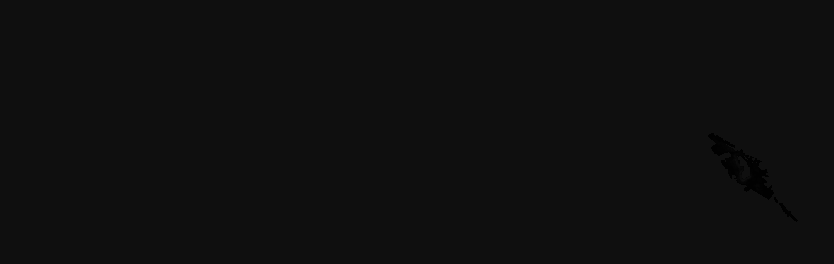

Supplement: S3 File — Files are in TIFF format to be displayed in ESRI ArcGIS 10.2 or higher. The attribute table for each TIFF file contains a column (PERCENT_RE) identifying the percentage of respondents in that time period fishing that 1.5 x 1.5 km grid cell (i.e., S2 Fig, S3 Fig). All files are projected in Alaska Albers coordinate system (NAD 1983–2011 Alaska Albers, WKID: 102966, Authority: ESRI). (ZIP) [file pone.0179584.s010.zip › S10_lingcod_rockfish/Sitka_lingcod_rockfish_2010_2015.tif]

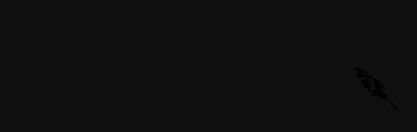

Supplement: S3 File — Files are in TIFF format to be displayed in ESRI ArcGIS 10.2 or higher. The attribute table for each TIFF file contains a column (PERCENT_RE) identifying the percentage of respondents in that time period fishing that 1.5 x 1.5 km grid cell (i.e., S2 Fig, S3 Fig). All files are projected in Alaska Albers coordinate system (NAD 1983–2011 Alaska Albers, WKID: 102966, Authority: ESRI). (ZIP) [file pone.0179584.s010.zip › S10_lingcod_rockfish/Sitka_lingcod_rockfish_2010_2015.tif.ovr]
