# Supplementary material for: Evaluating patterns and drivers of spatial change in the recreational guided fishing sector in Alaska
Source: PLoS One. 2017 Jun 20;12(6):e0179584. doi: 10.1371/journal.pone.0179584 (PMC5478146; doi:10.1371/journal.pone.0179584)
Supplement: S1 Text — After acquiring informed consent, the interview team asked a series of questions to the participant on charter fishing experience, charter business information, and fishing locations. (DOCX) [file pone.0179584.s011.docx]

**Experience and personal history** [Researcher fills out]

1. In Alaska, what fisheries have you participated in? Mark more than one if applicable.

□ Commercial fishing

Which years: ____________________________________________________________

Target species: ___________________________________________________________

Methods / gear type(s) used: ________________________________________________

Approx. days per year and changes over time:

□ Recreational fishing - Unguided

Which years: ____________________________________________________________

Target species: ___________________________________________________________

Methods / gear type(s) used: _____________________________________

Approx. days per year and changes over time:

□ Subsistence fishing

Which years: ____________________________________________________________

Target species: ___________________________________________________________

Methods / gear type(s) used: ________________________________________________

Approx. days per year and changes over time:

□ Other, please specify: ___________________________________________

Which years: ____________________________________________________________

Target species: ___________________________________________________________

Method(s) used: __________________________________________________________

Approx. days per year and changes over time:

□ Recreational fishing – Charter fishing (as an operator only)

Which years: ____________________________________________________________

Target species: ___________________________________________________________

Methods / gear type(s) used: ________________________________________________

Approx. days per year and changes over time:

**Background information on charter business** [Respondent fills out]

1. How would you characterize your involvement in the charter fishery? (Circle all that apply)
2. Charter owner/operator, single vessel
3. Charter owner/operator, multi-vessel owner
4. Charter business / lodge owner
5. Employee (hired as captain)
6. Independent contractor (hired for services as captain and boat)
7. What best describes the majority of your business activity? (Circle all that apply)
8. Fishing only
   - 1. “Half day” trip
     2. “Three-quarter day” trip
     3. “Full day” trip
     4. “Overnight” trip
     5. “Multi day” trip
9. Combination fishing and dedicated eco-tour/wildlife-viewing
10. Combination fishing and hunting
11. How many and what type of boats does this operation have?
12. How many Charter Halibut Permits does this operation have?
13. What is your position within this operation? (examples: lead skipper, owner-operator)
14. What months do you participate in this charter operation?

**Spatial changes** [Respondents fill out]

1. On the maps provided, mark the areas that you target halibut and each of the other species for each decade you have participated in the charter sector. [Respondents will be given a different color for each species.]

Currently, what are the areas you target the following species:

Halibut - green

Salmon - orange

Lingcod - yellow

Rockfish - pink

Other -

Have your fishing areas changed since you first started charter fishing? Why?

Can you recommend anyone else to interview?
